# Evolutionary differences in gene loss and pseudogenization among mycoheterotrophic orchids in the tribe Vanilleae (subfamily Vanilloideae)

**DOI:** 10.3389/fpls.2023.1160446

**Published:** 2023-03-22

**Authors:** Lisi Zhou, Tongyao Chen, Xiandan Qiu, Jinxin Liu, Shunxing Guo

**Affiliations:** Institute of Medicinal Plant Development, Chinese Academy of Medical Sciences & Peking Union Medical College, Beijing, China

**Keywords:** mycoheterotrophic, Vanilloideae, plastome, gene loss, rearrangement, IR/SC boundaries

## Abstract

**Introduction:**

*Galeola lindleyana* is a mycoheterotrophic orchid belonging to the tribe Vanilleae within the subfamily Vanilloideae.

**Methods:**

In this study, the *G. lindleyana* plastome was assembled and annotated, and compared with other Vanilleae orchids, revealing the evolutionary variations between the photoautotrophic and mycoheterotrophic plastomes.

**Results:**

The *G. lindleyana* plastome was found to include 32 protein-coding genes, 16 tRNA genes and four ribosomal RNA genes, including 11 pseudogenes. Almost all of the genes encoding photosynthesis have been lost physically or functionally, with the exception of six genes encoding ATP synthase and *psaJ* in photosystem I. The length of the *G. lindleyana* plastome has decreased to 100,749 bp, while still retaining its typical quadripartite structure. Compared with the photoautotrophic Vanilloideae plastomes, the inverted repeat (IR) regions and the large single copy (LSC) region of the mycoheterotrophic orchid’s plastome have contracted, while the small single copy (SSC) region has expanded significantly. Moreover, the difference in length between the two *ndh*B genes was found to be 682 bp, with one of them spanning the IRb/SSC boundary. The Vanilloideae plastomes were varied in their structural organization, gene arrangement, and gene content. Even the *Cyrtosia septentrionalis* plastome which was found to be closest in length to the *G. lindleyana* plastome, differed in terms of its gene arrangement and gene content. In the LSC region, the *psb*A, *psb*K, *atp*A and *psa*B retained in the *G. lindleyana* plastome were missing in the *C. septentrionalis* plastome, while, the *mat*K, *rps*16, and *atp*F were incomplete in the *C. septentrionalis* plastome, yet still complete in that of the *G. lindleyana*. Lastly, compared with the *G. lindleyana* plastome, a 15 kb region located in the SSC area between *ndh*B*-rrn*16S was found to be inverted in the *C. septentrionalis* plastome. These changes in gene content, gene arrangment and gene structure shed light on the polyphyletic evolution of photoautotrophic orchid plastomes to mycoheterotrophic orchid plastomes.

**Discussion:**

Thus, this study’s decoding of the mycoheterotrophic *G. lindleyana* plastome provides valuable resource data for future research and conservation of endangered orchids.

## Introduction

The Orchidaceae, one of the two largest families of flowering plants, are unique in features such as their labellum, stamen, pollen block and dust-like seeds (Chase et al., 2015). They include approximately 28,000 species in 736 genera (Christenhusz and Byng, 2016), and are distributed throughout the world. Molecular phylogenetic research has revealed the Orchidaceae to be a monophyletic group, divided into five subfamilies, namely Apostasioideae, Cypripedioideae, Vanilloideae, Orchidoideae and Epidendroideae (Chase et al., 2015; Li et al., 2016). Among these, the Vanilloideae, Orchidoideae and Epidendroideae subfamilies include both photoautotrophic and mycoheterotrophic orchids (Merckx, 2013), with 232 mycoheterotrophic species in 43 genera. This feature makes them ideal material for research related to trophic transformation and gene structure change. Molecular evidence has shown that the rate of evolution in the Vanilloideae subfamily has variously accelerated and slowed down (Wang et al., 2018), and it is the first independent clade to include mycoheterotrophic species after the Apostasioideae subfamily (Kim et al., 2020). Therefore, further in-depth investigation of the Vanilloideae subfamily plastomes can provide more abundant evidence for the analysis of the genetic evolution mechanism, as well as establish a molecular basis of trophic-type changes in orchids in their early evolutionary stage.

Photosynthesis is known to be the primary means by which plants obtain their nutrients. However, limited by factors such as challenging living conditions or oxidative stress, plants have also adaptively evolved alternative survival strategies such as mycoheterotrophy and parasitism (Leake, 1994; Bidartondo, 2005; Merckx and Freudenstein, 2010; Westwood et al., 2010; Delannoy et al., 2011; Merckx, 2013; Wicke et al., 2016). While such changes in the survival strategies and vegetative organs of plants occurred, research has shown that traces of gene variation and selection remained in their plastomes (Wicke et al., 2011; Bromham et al., 2013; Petersen et al., 2015; Wicke et al., 2016). The transition from autotrophic to parasitic was accompanied by the loss of photosynthesis and housekeeping plastid genes (Wolfe et al., 1992; Funk et al., 2007), as well as the function, size (Lohan and Wolfe, 1998) and transcriptional association with essential genes (Wicke et al., 2013), all of which are linked to the retention or lost of the gene in the plastids of non-photosynthetic plants. Therefore, based on data published regarding the evolutionary transition from phototrophic to parasitic plastomes, an overall trend prediction for progressive gene loss has been proposed (Barrett and Davis, 2012; Barrett et al., 2014a; Bellot and Renner, 2016; Naumann et al., 2016; Wicke et al., 2016). In this hypothesis, the plastid genome reduction process is divided into the following four stages: 1) The loss of the NADH dehydrogenase-like (NDH-1) complex, generally regarded as the earliest loss of plastid-encoded genes; 2) This stage was followed by pseudogenization and the loss of photosynthetic genes, the deprivation of photosynthetic function and the removal of selection pressure to retain photosynthetic plastid genes; 3) Subsequently, the loss of genes for the plastid-encoded subunits of RNA polymerase and photosynthetic enzymes with minor functions (Rubisco and ATP synthase), the relative timing of which had an asynchronous and comparatively wide window; and 4) The delayed loss of the five core non-bioenergetic genes (especially *trn*E and *acc*D, which encode glutamyl tRNA and acetyl-CoA carboxylase subunits, respectively), observed only in fully parasitic plastomes with large-scale gene loss. The range of changes of mycoheterotrophic plastomes is similar to that of parasitic species (Barrett et al., 2014b; Lam et al., 2015; Lam et al., 2016; Kim et al., 2019; Kim et al., 2020), however, in the evolution of plastid genomes in mycoheterotrophic orchids belonging to the same tribe, gene variation and loss have been specific rather than convergent. Feng et al. (2016), for example, found that the transitions to a fully mycoheterotrophic lifestyle evolved independently at least three times during the evolution of the tribe Neottieae. Despite the general trend of plastid degradation was similar during the transition from autotrophic to mycoheterotrophic and from autotrophic to parasitic, highly lineage-specific plastome degeneration still occurred.

The Vanilloideae subfamily includes 14 genera and 245 species (Chase et al., 2015) with various lifestyles, including epiphytic and terrestrial. Only nine orchid plastomes have thus far been reported, distributed among four genera and two tribes (Cameron, 2009; Lin et al., 2015; Amiryousefi et al., 2017; Niu et al., 2017; Kim et al., 2019; Kim et al., 2020). Of the 14 Vanilloideae subfamily heterotrophs in five genera, only three species in two genera have been reported. Due to the lack of decoded mycoheterotrophic Vanilloideae plastomes, the study of genetic variation in plastomes during the lifestyle transition from photoautotrophic to mycoheterotrophic has thus far been insufficient, and the diversity of evolutionary models has not been verified. Thus, this study sequenced, assembled, and annotated the mycoheterotrophic *G. lindleyana* plastome, subsequently comparing it with the plastomes of previously researched photosynthetic and heterotrophic orchids in the same subfamily, in order to explore the genetic variations occurring in plastomes that transitioned from photoautotrophic to mycoheterotrophic.

## Material and methods

### Plant materials, DNA extraction and high-throughput sequencing

**Figure 1:**
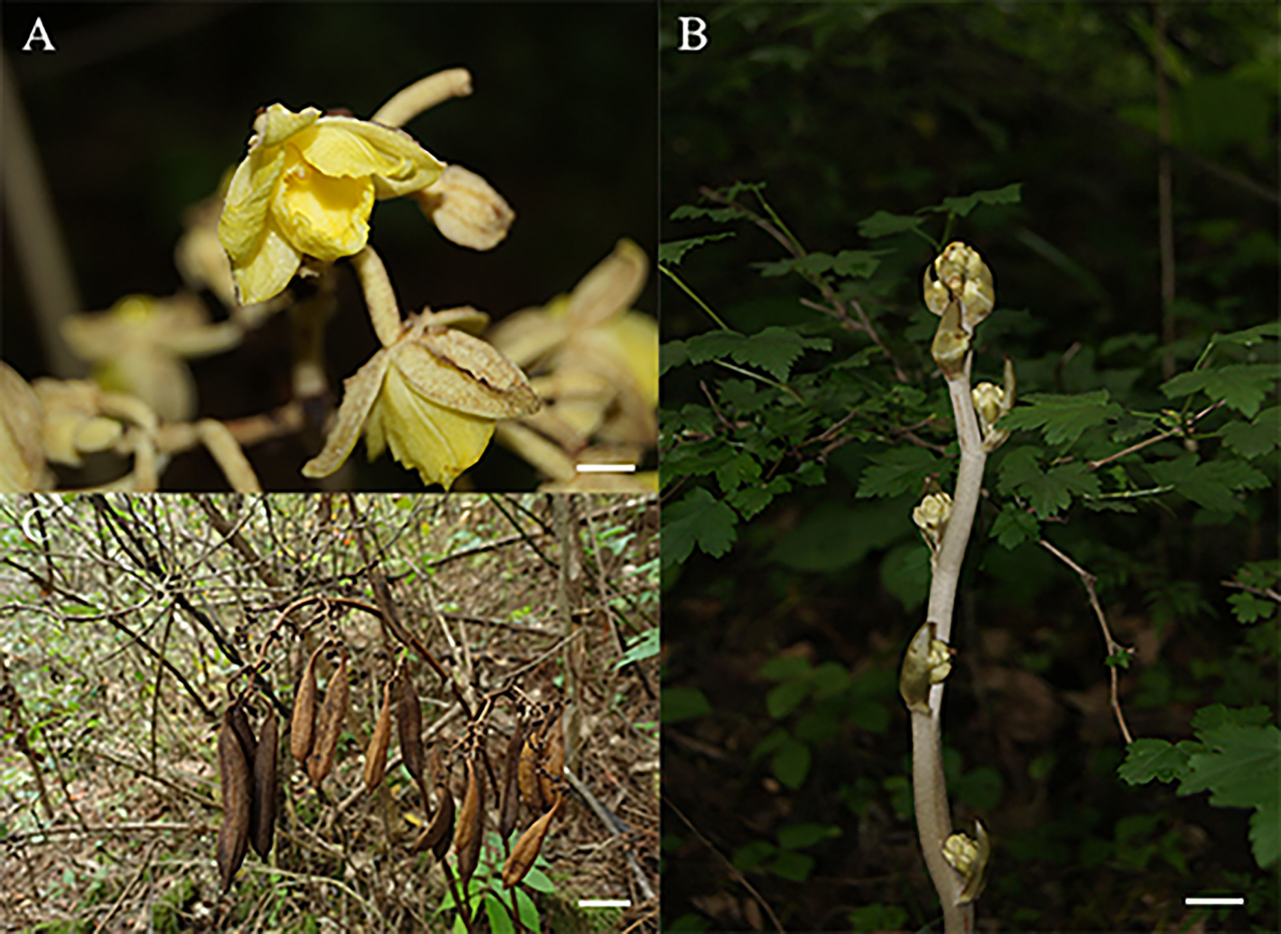
Morphological characteristics of *G. lindleyana:* **(A)** Flower, scare bar = 1cm; **(B)** stem, scare bar = 1cm; **(C)** capsule, scare bar = 5cm.

Since the *G. lindleyana* plant does not have typical leaf organs, its root was used to obtain its chloroplast genome sequence. The root of *G. lindleyana* (**Figure 1**) was collected from Yuexi in Anhui Province, China (30.84°N; 116.34°E) at an altitude of 1130 m on October 2, 2020. This plant was identified morphologically, and its voucher specimen deposited in the Institute of Medicinal Plant Development, Chinese Academy of Medicinal Sciences.

Fresh root samples were ground into fine powder with liquid nitrogen in a mortar, and then used to extract the genomic DNA using a Plant Genomic DNA Extraction Kit (Tiangen Biotech (Beijing) Co., Ltd., China). The DNA concentration and the ratios of A260/A280 and A260/A230 were measured using a Thermo Scientific NanoDrop 2000 ultra-micro spectrophotometer (Thermo Fisher Scientific Inc., MA, USA). Following the construction of a 270 bp PCR-free library, the whole genomic DNA of *G. lindleyana* was sequenced using the Illumina NovaSeq 6000 platform *via* shotgun sequencing.

### Plastome assembly and annotation

The resulting raw high-throughput sequencing reads were trimmed and filtered using Trimmomatic v0.38 (Bolger et al., 2014). The complete *G. lindleyana* chloroplast genome was assembled using the GetOrganelle(v1.7.7.0) toolkit (Jin et al., 2020), and four junction regions between IRs and LSC/SSC were subsequently confirmed *via* polymerase chain reaction (PCR) amplifications and Sanger sequencing using the primers listed in **Table S1**. The genome sequence was automatically annotated using the CPGAVAS2 integrated web server (Shi et al., 2019), and then manually edited using the Apollo editor according to separate BLASTN results comparing the CDS and protein sequences of 238 previously published plastid genomes in the Orchidaceae family.

### Genome comparison, divergence and phylogenetic analysis

Genome features, such as size, LSC region, SSR region, IR region, GC content and unique genes, were analyzed or counted using a local Python script. SSRs were predicted *via* MISA(http://pgrc.ipk-gatersleben.de/misa/) microsatellite finder (Beier et al., 2017);, and the tandem repeat sequences were found using a tandem repeats finder (TRF) (Benson, 1999). SC-IR junction regions were described *via* IR Scope among six Vanilleae species (Amiryousefi et al., 2018), while chloroplast genome comparison was completed using mVISTA (http://genome.lbl.gov/vista/mvista/submit.shtml) with the annotation of four Vanilleae species as references (Frazer et al., 2004). Pairing sequence alignments of the cp genomes were performed using Mummer v3.23 with the six Vanilleae species and five mycoheterotrophic orchids (Kurtz et al., 2004). Synonymous codon usage and RSCU were analyzed using the CodonW v1.4.2 (Sharp and Li, 1987). The plastome sequence gene data of 59 Orchidaceae species were also used to construct a maximum likelihood (ML) phylogenetic tree, using five species (*Allium cepa, Eustrephus latifolius, Iris gatesii, Iris sanguinea* and *Fritillaria hupehensis*) as out groups. A total of 79 CDS genes were extracted and aligned using MAFFT software v7.515 (Katoh et al., 2002), and these alignments were subsequently concatenated into a single length of 71, 597 bp. The missing data of CDS genes were treated as insertions/deletions, and filled the alignments with dashs. Concatenated alignments were then used to perform the JModelTest. Finally, an ML tree was constructed *via* RAxML v8.2.12 (Stamatakis, 2014) with a GTR+G+I model with 1000 bootstrap replicates.

## Results

### Features of *G. lindleyana* cpDNA

**Figure 2:**
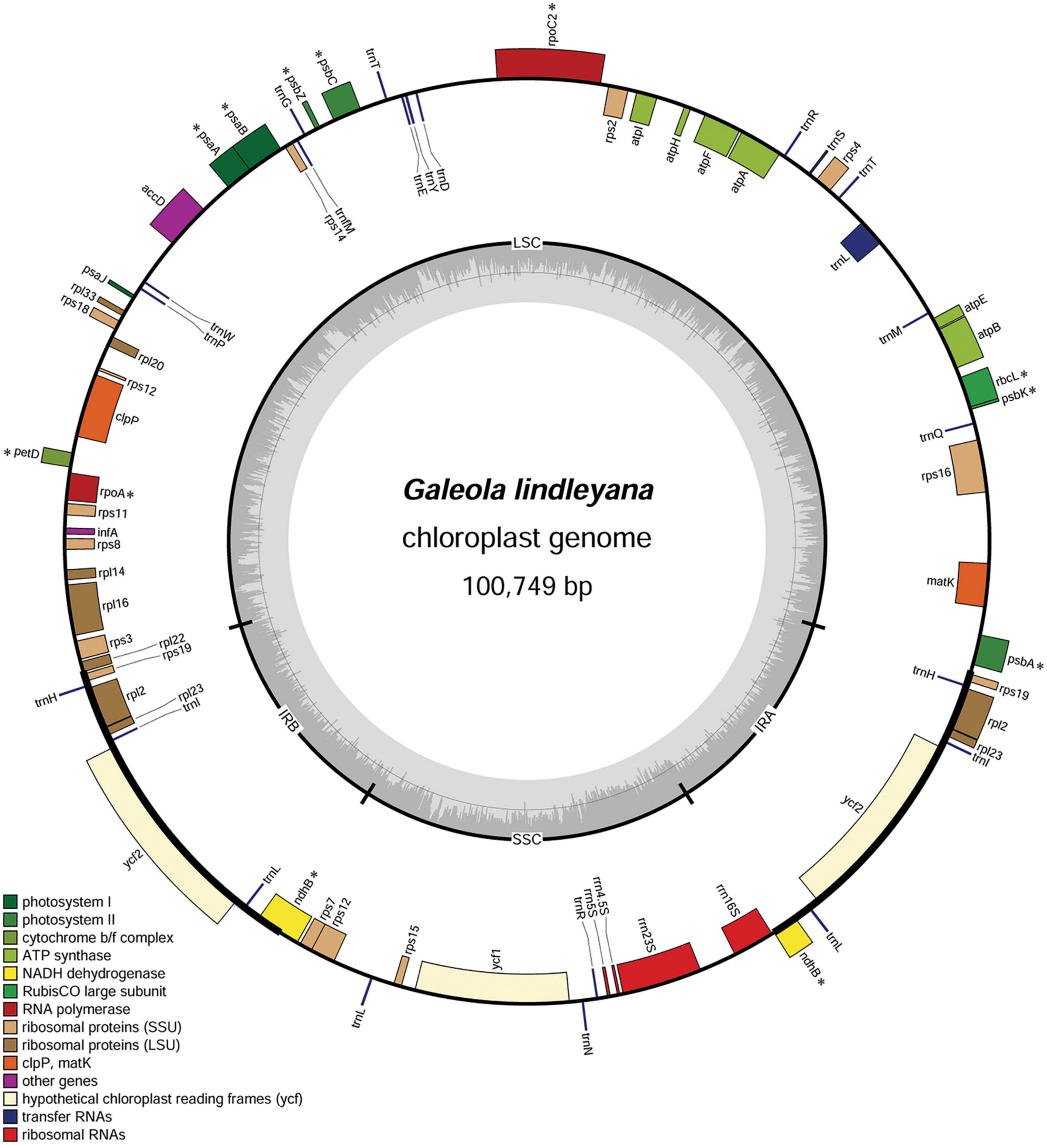
Chloroplast genome map of *G. lindleyana.* Genes inside the circle are transcribed clockwise, whereas those outside are transcribed counterclockwise. The darker gray in the inner circle corresponds to the GC content, while the lighter gray corresponds to the AT content. Pseudogenes are marked with an asterisk (*).

A total of 43 million pair-end reads were produced with 5.81 Gb of clean data. Data from all of the reads were deposited in the NCBI Genbank under accession number MW528436. The complete plastome was found to be 100,749 bp (**Figure 2**), and displayed a typical quadripartite structure, including a pair of inverted repeat regions (IR; 11,607 bp) separated by large single copy (LSC; 59,493 bp) and small single copy (SSC; 18,042 bp) regions, covering 11.5%, 59.1% and 17.9% in the plastome, respectively (**Table 1**). The total GC content in the whole *G. lindleyana* plastome was 34.36%, with the LSC region containing the lowest amount of GC contents (31.24%) compared to those of the SSC (38.91%) and IR (38.80%) regions.

**Table 1:** Chloroplast genome features of *G. lindleyana* and its closely related species in the Vanilleae tribe.

Taxon Name	Life style	Nutritional mode	Genome Size/bp	LSC Length/bp	SSC Length/bp	IR Length/bp	GC Content/%	No. of coding genes	No. of tRNAs
*Galeola lindleyana*	Terrestrial	Mycoheterotrophic	100749	59493	18042	11607	34.4	32	16
*Cyrtosia septentrionalis*	Terrestrial	mycoheterotrophic	96859	58085	17946	10414	34.8	41	25
*Lecanorchis japonica*	Terrestrial	mycoheterotrophic	70498	28197	14493	13904	30.4	25	7
*Lecanorchis kiusiana*	Terrestrial	mycoheterotrophic	74084	30824	14118	14571	30.0	25	8
*Vanilla aphylla*	Epiphyte	Photosynthetic	150165	87379	3354	29716	35.0	65	29
*Vanilla madagascariensis*	Epiphyte	Photosynthetic	151552	87490	1254	31404	34.6	71	29
*Vanilla planifolia*	Epiphyte	Photosynthetic	148011	86358	2037	29808	35.4	72	30
*Vanilla pompona*	Epiphyte	Photosynthetic	148009	86358	2037	29807	35.4	72	30

A total number of 63 genes were encoded in the plastome, including 32 protein coding genes, 16 tRNA, and 4 rRNA genes, including 11 pseudogenes (**Table 2**). The protein-coding genes included 12 genes encoding small subunits of ribosome (*rps*2, 3, 4, 7, 8, 11, 12, 14, 15, 16, 18 and 19), eight genes encoding large subunits of ribosome (*rpl*2, 14, 16, 20, 22, 23 and 33), six genes encoding ATP synthase (*atp*A, B, E, F, H, and I), two genes encoding conserved open reading frames (*ycf*1 and 2), only one gene related to photosystem I (*psa*J), and four genes encoding subunits of acetyl-CoA-carboxylase (*acc*D), protease (*clp*P), translational initiation factor (*inf*A) and maturase (*mat*K), respectively. Among these unique genes, four (*atp*F, *rpl*2, *rps*16 and *trn*L) were found to have one intron each, while two genes (*clp*P and *rps*12) comprised two introns each. Almost all of the genes encoding photosynthesis had undergone pseudogenization, with the exception of six genes encoding ATP synthase and *psa*J in photosystem I, which were still intact.

**Table 2:** Gene composition in *G. lindleyana* chloroplast genome.

Gene functions	Gene set	Gene
Photosynthesis	Photosystem I	*psa*A* , psa*B* *, *psa*J**
	Photosystem II	*psb*A* , psb*C* , psb*K* , psb*Z* *
	Cytochrome b/f complex	*pet*D**
	ATP synthase	*atp*A, *atp*B, *atp*E, *atp*F*, *atp*H, *atp*I
	NADH-dehydrogenase	*ndh*B×2
	Rubisco	*rbc*L
Self replication	Large subunit of ribosome	*rpl*14, *rpl*16, *rpl*2×2*, *rpl*20, *rpl*22, *rpl*23×2, *rpl*33
	Small subunit of ribosome	*rps*11, *rps*12**, *rps*14, *rps*15, *rps*16*, *rps*18, *rps*19×2, *rps*2, *rps*3, *rps*4, *rps*7, *rps*8
	DNA dependent RNA polymerase	*rpo*A, *rpo*C2
Other gene	rRNA genes	*rrn*16S, *rrn*23S, *rrn*4.5S, *rrn*5S
	tRNA genes	*trn*D, *trn*E, *trn*G, *trn*H, *trn*I, *trn*L*, *trn*M, *trn*N, *trn*P, *trn*Q, *trn*R, *trn*S, *trn*T, *trn*W, *trn*Y, *trnf*M
	Subunit of Acetyl-CoA-carboxylase	*acc*D
	Protease	*clp*P**
	Translational initiation factor	*inf*A
	Maturase	*mat*K
Unkown	Conserved open reading frames	*ycf*1, *ycf*2×2

“×2” indicates that the number of repeat units is two; One or two asterisks following genes indicate one or two contained introns, respectively. Pseudogenes are marked with an underscore.

In this plastome the most frequently used codon was AAA (n=1374) followed by AAT (n=1032), encoding lysine and asparagine, respectively. The least frequently used codon was stop codon TGA (n=31).

### Codon usage

Codon usage patterns of the coding sequences for *G. lindleyana* were calculated based on the relative synonymous codon usage (RSCU) value. All protein-coding genes in *G. lindleyana* plastome were encoded by 9749 codons (**Figure S1**). A total of 61 codons encoded 20 amino acids, with three stop codons. There were 32 preferred and 32 non-preferred codon usages. Among them, the most abundant amino acid was leucine, with 962 codons (9.87% of total), followed by isoleucine with 838 codons (8.60% of total) (**Figure S1A**), while stop codons were the fewest, at just 31 (0.32% of total). Almost all amino acids had more than one synonymous codon, with the exceptions of tryptophan and methionine. Arginine, serine and leucine had the most synonymous codon usages.

The RSCU value was used to evaluate the synonymous codon bias, and codons with greater RSCU values were preferred in each case (**Figure S1B**). The most preferred codon was found to be AGA encoding amino acid arginine (Arg), with 1.99 RSCU, followed by UUA encoding leucine (Leu) and UCU encoding serine (Ser) with 1.79 and 1.73 RSCU, respectively. By contrast, the lowest frequency codon was found to be CAC encoding histidine (His) with 0.32 RSCU, followed by CGC encoding arginine (Arg) with 0.37 RSCU. With the exceptions of the leucine encoded by UUG and serine encoded by UCC, amino acid codons (RSCU > 1) in the *G. lindleyana* plastome preferentially showed A- or U-endings, and non-preferred codon usages ended with base C or G, corresponding to the previously mentioned results that were calculated based on protein-coding sequences.

### Simple sequence and tandem repeat analyses

Simple sequence repeats (SSRs, also known as microsatellites) and tandem repeats (TRs) in the *G. lindleyana* plastome were surveyed in this study. A total of 41 SSRs were detected, following strict performance parameters (unit_size/min_repeats): 1/10 (mononucleotides ≥ 10 nt), 2/6 (dinucleotides ≥ 6 repeats), 3/5, 4/5, 5/5 and 6/5 (**Table S1**). Analysis included 29 mononucleotide repeats, nine dinucleotide repeats and two trinucleotide repeats, with only one hexa-nucleotide SSR identified. No tetra- or penta-nucleotide SSRs were found. The majority of SSRs were mononucleotides repeats (70.7%) in which base T and A were the primary elements with a proportion of 68.3%, only one C motif and no G motifs. The T-repeat unit was found to be most abundant in this study, with a total of 16, while the hexa-nucleotide SSR was repeated most frequently at 23 times in total.

There were 116 copy number variations (CNVs) of TR units (**Table S1**) in the *G. lindleyana* plastome ranging in length from 7 to 63 bp, with those of 16 and 18 bp most abundant (12), followed by those of 12 bp (10), and then those of 13 bp (7) and 24 bp (7). Most of the 116 CNVs were found to be present in intergenic regions, and only nine were present in the genic regions of the *acc*D, *ycf*1 and *ycf*2 genes, and the *psb*C pseudogene. The TR units appeared more frequently in the LSC regions (81.0%) than in the SSC (12.9%) and IR (6.0%) regions. CNVs of various TR units related to indel polymorphism were also identified in the *G. lindleyana* plastome.

### Junctions of inverted repeats and single copy regions

**Figure 3:**
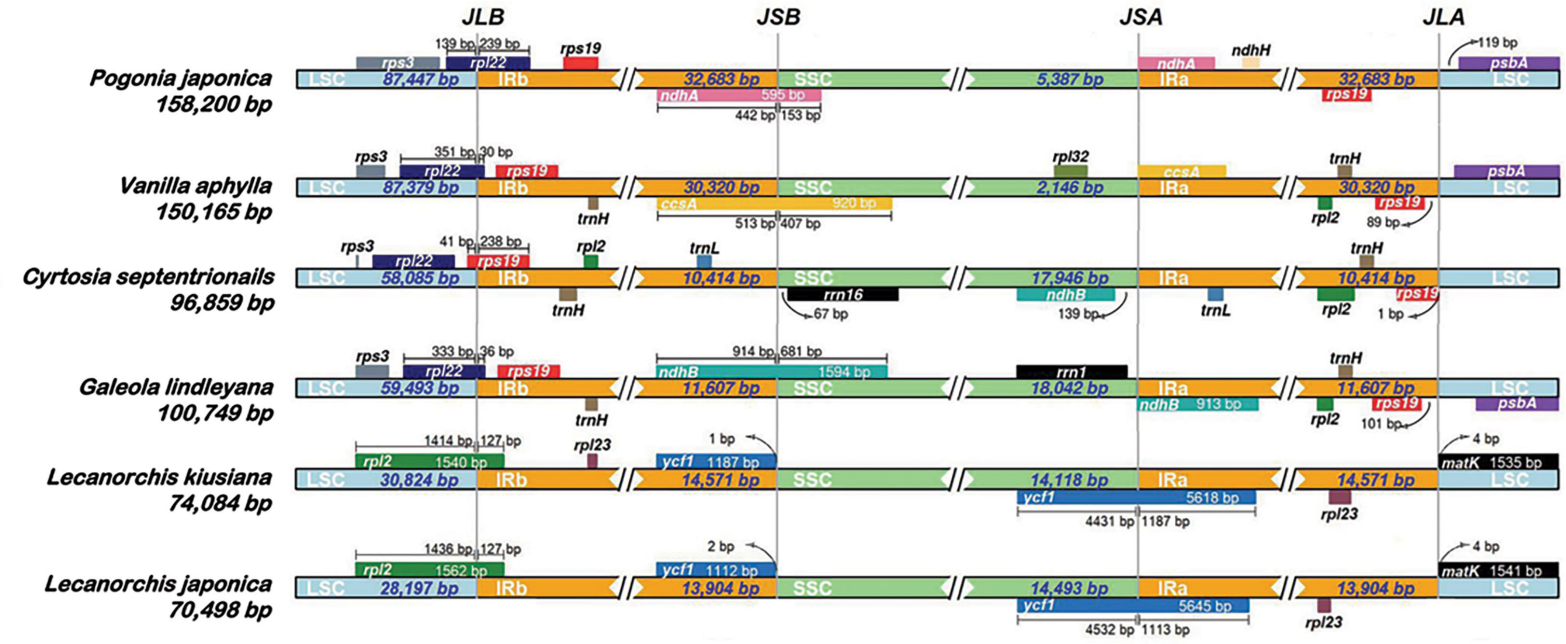
Comparisons of LSC, IRb, SSC and IRa border regions in six species of Vanilleae.

A comprehensive assessment of the four junctions (J_LA_, J_LB_, J_SA_ and J_SB_) between the two IR regions and the two single copy regions (LSC and SSC) of six species in the subfamily Vanilloideae was also performed, and the results are presented in **Figure 3**. Evidence of substantial expansion and contraction in both the IR regions and the two single copy regions were detected, with the IR regions ranging from 32,683 bp in *Pogonia japonica* to 10,414 bp in *Cyrtosia septentrionalis*; the LSC region ranging from 87,447 bp in *P. japonica* to 28,197 bp in *Lecanorchis japonica*; and the SSC region ranging from 18,042 bp in *G. lindleyana* to 2,146 bp in *Vanilla aphylla*.

Two junctions were conserved between the LSC region and the two IR regions in the same genus, but were variated in different genera. The distance between the J_LA_ border and *mat*K gene was found to be the same in all *Lecanorchis* plastomes. Similarly, the *rpl*2 gene that crossed J_LB_ in the two *Lecanorchis* plastomes was located in the LSC region but expanded to 127 bp to reach the IRb region. However, the J_LA_ and J_LB_ borders were variable in the other four species: The J_LB_ borders of *P. japonica, V. aphylla* and *G. lindleyana* plastomes were within the *rpl*22, with some of the sequences present in the LSC region, showing expansions of 239 bp, 30 bp and 36 bp to reach the IRb region, respectively, while the *C. septentrionalis* plastome exhibited LSC contraction, leading to the entire *rpl*22 in the LSC region.

The IR contraction in the *C. septentrionalis* plastome brought about the crossing of *rps*19 over the J_LB_ border, with 41 bp located in the LSC region and the entire *rps*19 in the IRb region of the *P. japonica, V. aphylla* and *G. lindleyana* plastomes. In addition, J_LA_ was found between *rps*19 and *psb*A in *P. japonica, V. aphylla* and *G. lindleyana* plastomes, although the *psb*A was not present at the J_LA_ border in *C. septentrionalis* plastome. The distances between *rps*19 and J_LA_ were found to be 1 bp in *C. septentrionalis* plastome, 89 bp in *V. aphylla* plastome and 101 bp in *G. lindleyana* plastome, respectively, all of which were longer than the distance of 119 bp between *psb*A and J_LA_ in *P. japonica* plastome.

In these six species belonging to the Vanilleae, more variations were found in the J_SA_ and J_SB_ than in the J_LA_ and J_LB_. In *Lecanorchis kiusiana* and *L. japonica* plastomes, the distance between *ycf*1 and J_SB_ was 1 bp and 2 bp, respectively, and the entire *ycf*1 was in the IRb region. However, the *ycf*1 in these two *Lecanorchis* species crossed the J_SA_ border at 4431 bp and 4532 bp located in SSC region and 1187 bp and 1113 bp in the IRa region in *L. kiusiana* and *L. japonica* plastomes, respectively. The genes around the J_SA_ and J_SB,_ and its location were varied in the other four species. In *C. septentrionalis* plastome*, rrn*16 and *ndh*B were located entirely in the SSC region, at 67 bp from the J_SB_ border and 139 bp from the J_SA_ border, respectively.On the J_SB_ border, the *ndh*A in *P. japonica*, *ccs*A in *V. aphylla* and *ndh*B in *G. lindleyana* crossed the IRb/SSC boundary, with 442 bp, 513 bp and 914 bp of each gene located in the IRb regions, respectively, and 153 bp, 407 bp and 1594 bp within the SSC regions, respectively. Moreover, the *ndh*A in *P. japonica*, *ccs*A in *V. aphylla* and *ndh*B in *G. lindleyana* were all at the beginning of the IRa regions, near the J_SA_ borders.

### Comparative analysis and sequence divergence analyses

**Figure 4:**
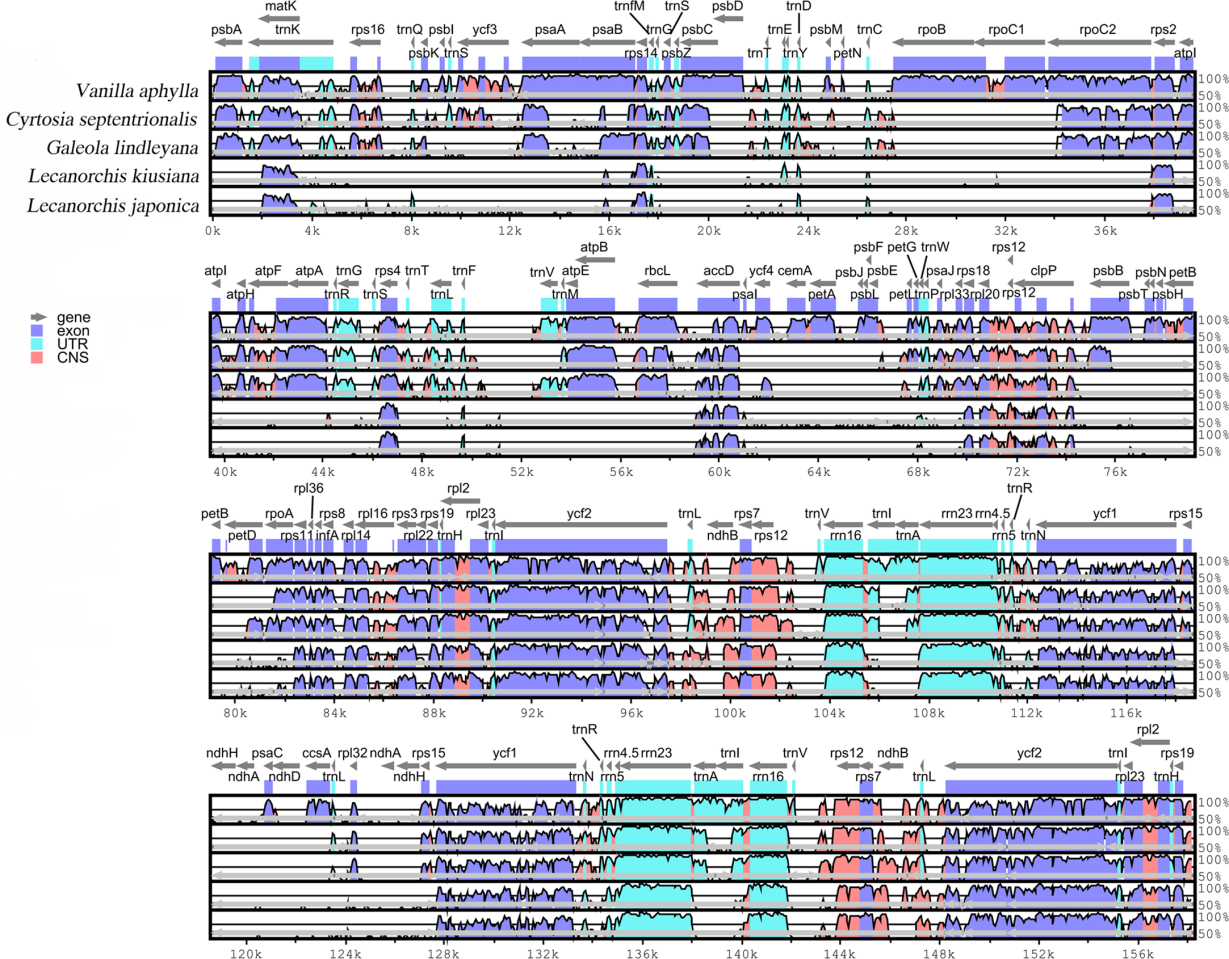
Global alignment of five chloroplast genomes of Vanilleae using mVISTA. Y-axis indicates the range of identity (50%-100%). Alignment was performed using *V. aphylla* as reference.

The differences in the four mycotrophic Vanilleae plastomes were evaluated using the phototrophic *V. aphylla* plastome as a reference, and the results are presented in **Figure 4**. Compared with the phototrophic species, the deletion of gene sequences in mycotrophic species was found to be significant. In *L. kiusiana* and *L. japonica*, all functional genes involved in photosynthesis have been lost. In *C. septentrionalis* and *G. lindleyana*, most genes of the photosystem were lost, while others, such as *psa*A, *psa*B and *psa*C, have been retained. Among these five species, the length of *G. lindleyana* and *C. septentrionalis* plastomes were found to be similar, but still showed significant differences in their functional gene loss and pseudo-genes. In the LSC area, the *psb*A, *psb*K, *atp*A and *psa*B retained by *G. lindleyana* were found to be missing in *C. septentrionalis*; while the *mat*K, *rps*16 and *atp*F present as pseudogenes in *C. septentrionalis* remain complete in *G. lindleyana*.

### Dynamic chloroplast genome structures of Vanilleae and three typical mycotrophic orchids

**Figure 5:**
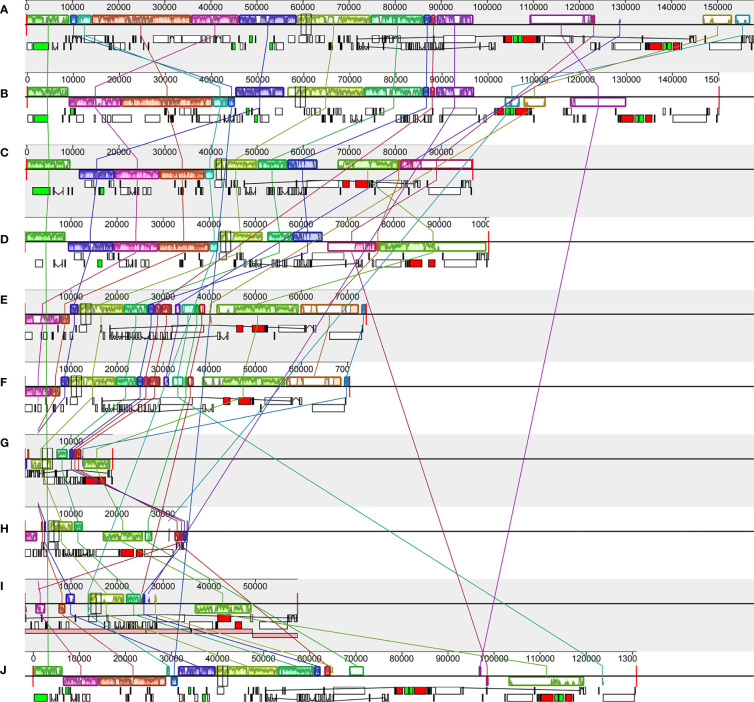
Comparisons of the complete chloroplast genome of six Vanilloideae species **(A)**. *P. japonica*; **(B)** *V. aphylla*; **(C)** *C. septentrionalis*; **(D)** *G. lindleyana*; **(E)** *L. kiusiana*; **(F)** *L. japonica*) and four typical mycoheterotrophic orchids **(G)**. *Epipogium roseum*; **(H)** *Gastrodia elata*; **(I)** *Rhizanthella gardneri*; **(J)** *Chamaegastrodia shikokiana*).

Gene block analysis, by which gene rearrangements are identified, was carried out using Mauve software among six Vanilleae orchids and four typical mycotrophic orchids (**Figure 5**). In the Vanilleae, rearrangement and inversion of genes appeared frequently, and even the phototrophic orchid plastomes were not fully colinear in all regions. In *P. japonica* and *V. aphylla*, approximately 37 kb of inversion occurred in the LSC area. However, compared with the other four mycotrophic orchid plastomes, gene loss and rearrangements of these two phototrophic orchid plastomes were limited. Among the Vanilleae, the species with the chloroplast genome most similar to that of *G. lindleyana* was *C. septentrionalis*, however, with a 15 kb inverted in the SSC area, between *ndh*B and *rrn*16S. Compared with *G. lindleyana*, the photosynthesis genes in the LSC regions of the *L. kiusiana* and *L. japonica* chloroplasts were absent, and the *rpl*2-*rps*19 in the IR area was inverted to the LSC area.

### Phylogenetic relationships

**Figure 6:**
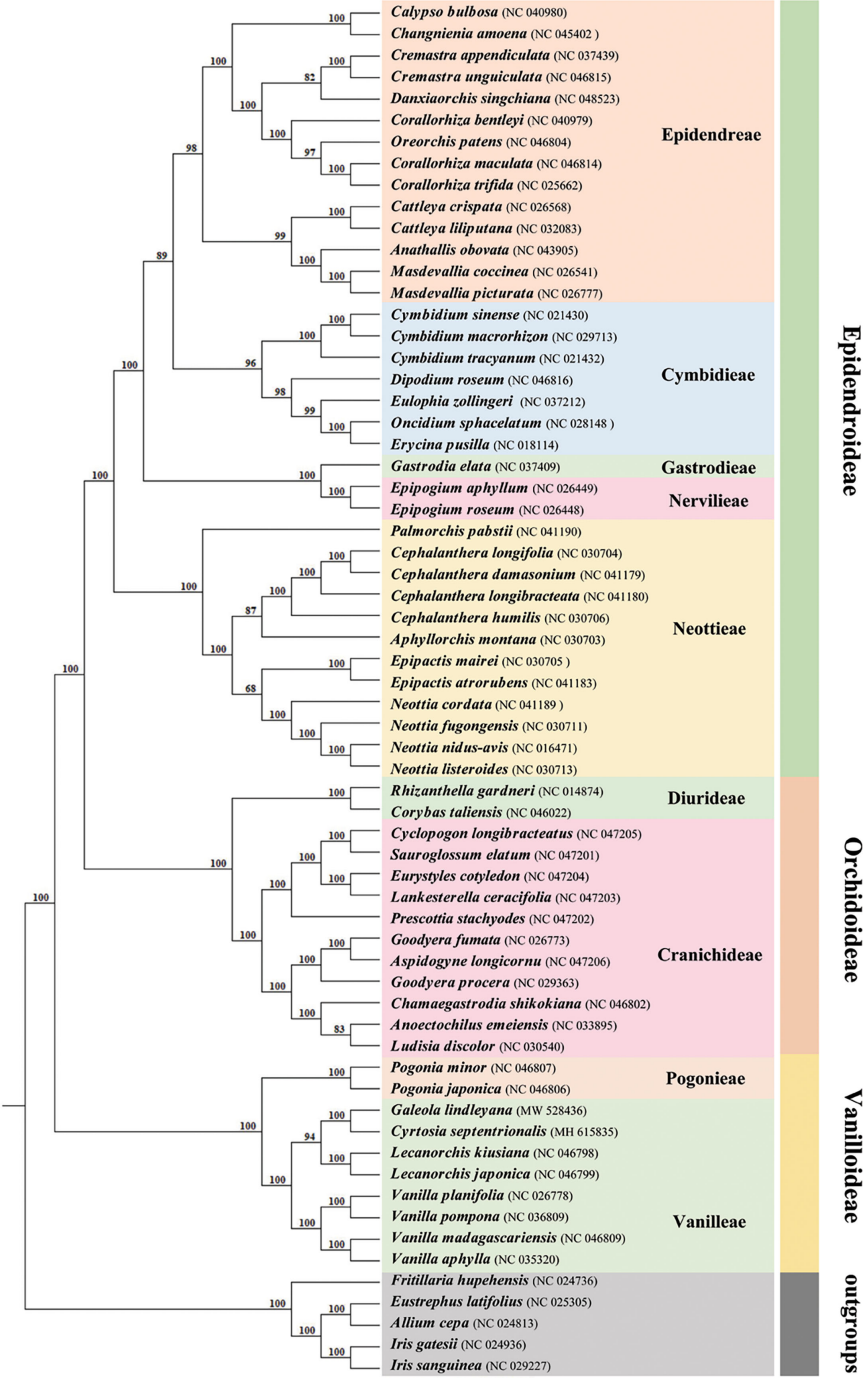
Phylogenetic tree for 59 Orchidaceae (with five non-Orchidaceae species as outgroups) using maximum likelihood (ML), based on the alignments of complete chloroplast genomes. Numbers at the nodes indicate bootstrap values from 1000 replicates.

In phylogenetic analysis, the Orchidaceae were divided into five subfamilies using the classification of Orchidaceae proposed by Chase et al. (2015). A total of 59 orchid plastomes from three of these subfamilies were employed to infer the phylogenetic relationship, using the maximum likelihood (ML) methods (**Figure 6**). Support values were consistently very high, except for the branch leading to the tribes Epidendreae, Neottieae and Cranichideae. Eight species belonging to Vanilleae formed a monophyletic group with high support. In the tribe Vanilleae, four mycoheterotrophic species were well distinguished from the photoautotrophic species and, within them, genus *Lecanorchis* was resolved as sister to the branch including *G. lindleyana* and *C. septentrionalis.* The respective branch support values for the branch including *G. lindle*yana, *C. septentrionalis* and genus *Lecanorchis* were lower than those for the branch including *G. lindle*yana and *C. septentrionalis*. The mycoheterotrophic plastomes belonging to the tribe Vanilleae are divided into two main clades, one of which is represented by genus *Lecanorchis*, while the other group contains two genera, namely *Galeola* and *Cyrtosia*. Among these three genera, the *Galeola* and *Cyrtosia* plastomes were found to have lost a similar length and number of genes, with a greater loss than that of *Lecanorchis*. Since only these four mycoheterotrophic chloroplast genomes have thus far been reported in Vanilleae, more sample data is needed to confirm any differences between the chloroplast genome loss strategies of the two genera species(*Galeola* and *Cyrtosia*).

## Discussion

### Rearrangements in plastomes

Although chloroplast genome structures are generally highly conserved, some do undergo changes during the long-term evolutionary process, subsequently appearing as either rearrangements or inversions. Rearrangements and inversions were detected in all chloroplast genomes of both the autotrophic and heterotrophic orchids in the Vanilloideae subfamily. In the *Pogonia* and *Vanilla* genera, rearrangement and inversion were seen in the LSC, IR and SSC regions, while these changes were seen only in the IR and SSC regions in the *Cyrtosia* and *Galeola* genera, and were confined to the LSC region in *Lecanorchis* genus. Rearrangements of the IR and SSC regions occur frequently and are thought to be the product of species evolution (Jansen and Palmer, 1987; Doyle et al., 1996; Chumley et al., 2006; Lee et al., 2007; Jansen et al., 2008; Ravi et al., 2008; Weng et al., 2014; Yan et al., 2017; Frailey et al., 2018; Roma et al., 2018). In *Pelargonium hortorum*, for example, the IR region of the chloroplast genome has extensively expanded, increasing in length to 76 kb, which is three times that of most common plants (Chumley et al., 2006). By contrast, the IR regions in Fabaceae and Cupressaceae have contracted significantly and even, in some, been completely lost (Saski et al., 2005; Hirao et al., 2008). In addition, the rearrangement of SSC regions has been detected in the chloroplast genomes of semi-parasitic species (Frailey et al., 2018), and the inversion of the SSC region, which appears to be a hallmark of the semi-parasitic group in the Orobanchaceae, has a chloroplast genome size similar to that of autotrophs. However, such rearrangement and inversion in all regions, as well as the initial contraction followed by expansion of the IR region, are rare, which suggests that the Vanilloideae subfamily may have been at the forefront of this evolutionary process. Changes in the nutrient types of the Vanilloideae subfamily, thus, closely related to the frequent occurrence of rearrangement and inversion.

### Variations in the IR/SC boundary

All chloroplast genomes have a typical four-part structure in the Vanilloideae subfamily, as do mycoheterotrophic orchids. However, differences have been found within the mycoheterotrophic orchids in the Orchidaceae subfamily, such as the loss of the IR region in *Gastrodia elata* plastome (Yuan et al., 2018; Kang et al., 2020; Park et al., 2020). The sizes and structures of chloroplast genomes in the Vanilloideae subfamily differ among the genera, and rearrangement and inversion occur in all four regions (Kim et al., 2015; Zeng et al., 2017). Among them, the longest chloroplast genome, at approximately 158,200 bp, is that of *P. japonica*, a member of the Pogonieae tribe, while the shortest, less than half the size at approximately 70,498 bp, is that of *L. japonica* belonging to the Vanilleae tribe. Such a dramatic difference in chloroplast genome length has also been found among the Neottieae tribe (Feng et al., 2016). However, some semi-heterotrophic species have also been found in the Orobanchaceae family, whose length of plastomes are similar to those of autotrophic species, suggesting that their plastomes are still in the early stages of their transition from autotroph to parasite (Wicke et al., 2013; Wicke and Naumann, 2018). The size of autotrophic orchid plastomes has been found to range from 147 kb to 151 kb, while those of mycoheterotrophic orchid plastomes fluctuate more widely, ranging from 70 kb to 100 kb. In mycoheterotrophic orchids, the plastomes of the genus *Lecanorchis* are smaller than 100 kb, while those of the genera *Cyrtosia* and *Galeola* are approximately 100 kb. Plastomes of the *Lecanorchis* orchid have not contracted significantly, possibly because the significant shrinkage of the LSC region is countered by the expansion of the SSC region. A similarly remarkable expansion of the SSC region to the IR region has also been reported in the Orobanchaceae family: Unexpectedly enlarged overall chloroplast genome lengths, attributable to the expansion of IR regions into SSC regions, have been noted in the semi-heterotrophic *Buchnera americana* as well as in three species belonging to the genus *Striga* in the same family (Frailey et al., 2018). Therefore, it is evident that the expansion of the SSC region in fully heterotrophic plants slowed down the pace of their plastome shrinkage, however, the reasons for this expansion still need to be explored. The expansion of SSC regions in the chloroplast genomes of semi-heterotrophic plants in the Orobanchaceae family and mycoheterotrophic orchids in the Vanilloideae subfamily is highly significant because it may indicate the evolution of key genes for energy metabolism in non-photosynthetic plants.

The IR/SC boundary and its nearby genes were analysed, among six Vanilloideae plastomes. and variations were found at all IR/SC boundaries. The shrinkage or expansion of the IR is known to lead to greater variability in the IR/SC boundary, and has been a recent hotspot in scientific research, especially in the Orchidaceae family(Wu et al., 2009; Wu et al., 2011; Sanderson et al., 2015). The LSC region has contracted in each of the six Vanilloideae plastomes, ranging from photosynthetic to mycoheterotrophic, with the LSC/IRB boundary gradually shrinking from *rpl*22 (*P. japonica*, *V. aphylla* and *G. lindleyana*) to *rps*19 (*C. septentrionalis*) and then to *rpl*2 (*L. kiusiana* and *L. japonica*). Moreover, the IR regions have contracted and then expanded from photosynthetic to mycoheterotrophic plastomes and the location of cross-boundary genes in the two boundaries was asymmetrical. The cross-boundary genes located in IRB/SSC and IRA/SSC boundaries changed gradually from *ndh*A (*P. japonica*) to *ccs*A (*V. aphylla*) to *ndh*B (*G. lindleyana*). However, the IR regions in *L. kiusiana* and *L. japonica* plastomes expanded,so that *ycf*1 regained its location in the IR regions. All Vanilloideae plastomes have genes across the IRB/SSC or IRA/SSC boundaries, with the exception of *C. septentrionalis.* Therefore, the cross-boundary genes are located in two IR regions and the SSC region simultaneously. In addition, the IRA/LSC boundary was found to have shrunk and then expanded. In *P. japonica*, *V. aphylla*, *G. lindleyana* and *C. septentrionalis* plastomes, the IRA region shrank gradually, and the *rps*19 moved to the IRA/LSC boundary gradually, while in *L. kiusiana* and *L. japonica* plastomes, the IRA region expanded until the *mat*K gene was located in the IRA/LSC boundary. These changes in the IR/SC boundaries are consistent with the two evolutionary routes in the Orchidaceae family (Yang et al., 2013; Luo et al., 2014): (1) The *ycf*1 gene expands continually into the IRA region, so the duplicated pseudogene *ψycf*1 fragment appearanced in the IRB region and overlapped with *ψndh*F. This evolutionary route was matched with changes in the heterotrophic orchid(*L. kiusiana* and *L. japonica*) plastomes. The SSC region expanded, causing the *ycf*1 to relocate in the IRA region; (2) The continuous movement of *ycf*1 to the SSC region resulted in the shorter length of the replicated *ycf*1 in the IRB region, which eventually moved completely into the SSC region, while the replicated *ycf*1 fragment disappeared in the IRB region. In this study, the transformation of plastomes from autotrophic (*P. japonica* and *V. aphylla*) to heterotrophic (*G. lindleyana* and *C. septentrionalis*) was found to be consistent with this hypothesis. The *ycf*1 was eventually located in the SSC region (*G. lindleyana* and *C. septentrionalis*). Changes in the spanning boundary genes were the major motivation for the contraction or expansion of IR regions(Goulding et al., 1996; Yang et al., 2010). Both of these hypotheses could well explain the changes at the IR/SC boundaries in the Orchidaceae family. Nevertheless, the evolutinonary origin and the chronological framework of cross-boundary gene changes were still largely unknown. Some researchers have shown that changes at the IR/SC boundaries could provide evidence for phylogenetic relationships (Gao et al., 2009; Wu et al., 2009; Wu et al., 2011; Yang et al., 2013; Downie and Jansen, 2015; Sanderson et al., 2015), however, in the Vanilloideae subfamily, the decoded plastomes were limited. In order to relate the changes at the IR/SC boundaries with phylogenetics, more Vanilloideae plastomes with various lifestyles should be collected and explored.

### Genes loss in plastid genomes and diversity of mycoheterotrophs in Vanilloideae orchids

In this study, the number of genes in the plastomes of each species in the Vanilloideae subfamily was found to be different. Pseudogenes and gene loss were present in the chloroplast genomes of all species. Most of the *ndh* gene family have been lost fully or partially, such as the remaining residual fragments of *ndh*A, *ndh*B, *ndh*D and *ndh*H. Rampant independent loss of the *ndh* gene family was a common feature across the Orchidaceae family (Kim et al., 2020) and, regardless of whether orchids were identified as mycoheterotrophic species or not, most of the *ndh* gene family were lost in the Vanilloideae subfamily orchid plastomes, with only the *ndh*B residual fragment still evident or a complete disappearance of the *ndh* gene (Kim et al., 2019; Kim et al., 2020). Assuming that the plastid genome degrades in a gradual manner, it may be also assumed the nonessential *ndh* gene pseudogenized before its fully physical deletion (Wicke et al., 2016; Wicke and Naumann, 2018). Pseudogenes could have been generated by premature codon termination and frameshift mutations (Balakirev and Ayala, 2003; Poliseno et al., 2010). The intron region of the *ndh*B gene in *G. lindleyana* plastome was found to have been shortened and was located at the IR/SC boundary, both favorable conditions for pseudogene generation. Moreover, the *ndh* gene family members were found to vary frequently and contained abundant repetitive sequences. Repeated sequences are considered to be one of the main reasons behind the rearrangement of the chloroplast genome (Yue et al., 2008). Therefore, the non-functionalization of the Vanilloideae subfamily chloroplast genome probably began with the pseudogenization and loss of the *ndh* gene family, as is consistent with the known evolutionary process of trophic changes (Wicke et al., 2013; Zeng et al., 2017). However, non-functionalization of the *ndh* gene and/or its fully physical loss was independent of trophic type in this species. Its polygenicity has been previously demonstrated in orchids (Barrett and Davis, 2012), carnivorous plants (Wicke et al., 2014) and even gymnosperms (Lin et al., 2010). In addition, Chang et al. (2006) found that *ndh*A, *ndh*F and *ndh*H in the *Phalaenopsis aphrodite* plastome had been transferred to its nuclear genome. Nonetheless, due to the lack of relevant nuclear genome data, it remains to be confirmed whether the *ndh* gene family has been transferred to its nuclear genome in the photosynthetic species of the Vanilloideae subfamily.

Currently, several models of chloroplast genome degeneration have been proposed to explain the physical or functional changes associated with the transition to a mycoheterotrophic lifestyle (Wicke et al., 2011; Bromham et al., 2013; Petersen et al., 2015; Wicke et al., 2016). In this study, photosynthetic plants were found to be in their initial stage of chloroplast genome degeneration, and the *ndh* gene appeared to be first pseudogenized and then lost entirely (Graham et al., 2017). During this period, reduced photosynthetic function begins with the loss of non-essential or stress-related genes (such as ndh genes) in half-heterotrophs. This is followed by pseudogenization and the loss of major photosynthesis-related genes (such as *pet*, *psa* and *psb* genes) and plastid-encoded polymerases. This was evident in this study, with both *G. lindleyana* and *C. septentrionalis* (Kim et al., 2019) found to be in this second stage of chloroplast genome degeneration. Among the photosynthesis-related genes, only *pet*N, *psa*J, *psb*M and *psb*Z remain in *C. septentrionalis*, while *G. lindleyana* no longer contains a complete gene of the photosynthesis-related genes and *pet*D, *psa*A, *psa*B, *psb*A, *psb*C, *psb*K and *psb*Z have all pseudogenized. The next stage of plastome degeneration ocurred in the edge of these shift from semi-mycoheterotrophic orchids to fully mycoheterotrophic orchids. Genes with extended or alternative functions, such as *atp* and *rbc*L, and non-essential housekeeping genes are lost after the transition to a non-photosynthetic or fully heterotrophic lifestyle, but prior to a plateau or slowing in the rate of gene loss. Herein, *L. kiusiana* and *L. japonica* (Kim et al., 2020) were found to be at this third stage of the plastome degeneration. In *L. kiusiana* and *L. japonica* plastomes, *psa* (5), *psb* (15), *pet* (6), *atp* (6), *rpo* (4) and *rbc*L were no longer evident, and the number of pseudogenes did not exceed three. Further loss of the chloroplast genome is followed by the deletion of other metabolic genes (such as *acc*D, *clp*P and *ycf*1/2), along with all other remaining housekeeping genes, including *trn*E. Thereafter, a stage of ‘total deletion’ is reached, in which the chloroplast genome is completely eradicated. At present, the data of mycoheterotrophic plastomes belonging to the Vanilloideae subfamily is limited, and it remains to be confirmed whether any species have undergone further plastome degeneration. However, it has been shown that the Vanilleae tribe has a very high evolutionary rate, and the plastome degeneration of mycoheterotrophic orchids in this tribe are various. Therefore, it is imperative to decode more chloroplast genome data of mycoheterotrophic plants belonging to the Vanilleae tribe.

## Data availability statement

The original contributions presented in the study are publicly available. This data can be found here: https://www.ncbi.nlm.nih.gov/nuccore/MW528436.1/.

## Author contributions

LZ, SG and JL conceived the research and experimental design. JL collected the plant material. LZ, TC and XQ performed the experiments and analyzed the data. LZ, SG and JL designed the draft manuscript. All authors approved the final draft for submission and take full public responsibility for the content of the manuscript.

## Supplementary material

Supplementary Figure 1 Codon frequencies and RSCU values of *G. lindleyana*: **(A)** Amino acid frequencies in protein-coding genes; **(B)** RSCU values of 20 amino acids and stop codons in 32 protein-coding genes.

## References

[B1] Amiryousefi A. Hyvönen J. Poczai P. (2017). The plastid genome of vanillon (*Vanilla pompona*, orchidaceae). Mitochondrial DNA Part B 2 (2), 689–691. doi: 10.1080/23802359.2017.1383201 33473949 PMC7800859

[B2] Amiryousefi A. Hyvönen J. Poczai P. (2018). IRscope: An online program to visualize the junction sites of chloroplast genomes. Bioinformatics 34, 3030–3031. doi: 10.1093/bioinformatics/bty220 29659705

[B3] Balakirev E. S. Ayala F. J. (2003). Pseudogenes: are they “junk” or functional DNA? Annu. Rev. Genet. 37, 123–151. doi: 10.1146/annurev.genet.37.040103.103949 14616058

[B4] Barrett C. F. Davis J. I. (2012). The plastid genome of the mycoheterotrophic *Corallorhiza striata* (Orchidaceae) is in the relatively early stages of degradation. Am. J. Botany 99, 1513–1523. doi: 10.3732/ajb.1200256 22935364

[B5] Barrett C. F. Freudenstein J. V. Li J. Mayfield-Jones D. R. Perez L. Pires J. C.. (2014a). Investigating the path of plastid genome degradation in early-transitional heterotrophic orchids, and implications for heterotrophic angiosperms. Mol. Biol. Evol. 31, 3095–3112. doi: 10.1093/molbev/msu252 25172958

[B6] Barrett C. F. Specht C. D. Jim L. M. Wm S. D. Zomlefer W. B. Davis J. I. (2014b). Resolving ancient radiations: Can complete plastid gene sets elucidate deep relationships among the tropical gingers (Zingiberales)? Ann. Botany 113, 119–133. doi: 10.1093/aob/mct264 24280362 PMC3864734

[B7] Beier S. Thiel T. Münch T. Scholz U. Mascher M. (2017). MISA-web: a web server for microsatellite prediction. Bioinformatics 33, 2583–2585. doi: 10.1093/bioinformatics/btx198 28398459 PMC5870701

[B8] Bellot S. Renner S. S. (2016). The plastomes of two species in the endoparasite genus *Pilostyles* (Apodanthaceae) each retain just fifive or six possibly functional genes. Genome Biol. Evol. 8, 189–201. doi: 10.1093/gbe/evv251 PMC4758247 26660355

[B9] Benson G. (1999). Tandem repeats finder: A program to analyze DNA sequences. Nucleic Acids Res. 27, 573–580. doi: 10.1093/nar/27.2.573 9862982 PMC148217

[B10] Bidartondo M. I. (2005). The evolutionary ecology of myco-heterotrophy. New Phytologist. 167, 335–352. doi: 10.2307/3694504 15998389

[B11] Bolger A. M. Lohse M. Usadel B. (2014). Trimmomatic: A flexible trimmer for illumina sequence data. Bioinformatics 30, 2114–2120. doi: 10.1093/bioinformatics/btu170 24695404 PMC4103590

[B12] Bromham L. Cowman P. F. Lanfear R. (2013). Parasitic plants have increased rates of molecular evolution across all three genomes. BMC Evol. Biol. 13, 1–11. doi: 10.1186/1471-2148-13-126 23782527 PMC3694452

[B13] Cameron K. M. (2009). On the value of nuclear and mitochondrial gene sequences for reconstructing the phylogeny of vanilloid orchids (Vanilloideae, orchidaceae). Ann. Bot. 104 (3), 377–385. doi: 10.1093/aob/mcp024 19251715 PMC2720648

[B14] Chang C. C. Lin H. C. Lin I. P. Chow T. Y. Chen H. H. Chen W. H.. (2006). The chloroplast genome of *Phalaenopsis aphrodite* (Orchidaceae): Comparative analysis of evolutionary rate with that of grasses and its phylogenetic implications. Mol. Biol. Evol. 23, 279–291. doi: 10.1093/molbev/msj029 16207935

[B15] Chase M. W. Cameron K. M. Freudenstein J. V. Pridgeon A. M. Salazar G. Van den Berg C.. (2015). An updated classification of orchidaceae. Botanical J. Linn. Soc. 177 (2), 151–174. doi: 10.1111/boj.12234

[B16] Christenhusz M. Byng J. (2016). The number of known plant species in the world and its annual increase. Phytotaxa 261, 201–217. doi: 10.11646/phytotaxa.261.3.1

[B17] Chumley T. W. Palmer J. D. Mower J. P. Fourcade H. M. Calie P. J. Boore J. L.. (2006). The complete chloroplast genome sequence of *Pelargonium hortorum*: Organization and evolution of the largest and most highly rearranged chloroplast genome of land plants. Mol. Biol. Evol. 11, 2175–2190. doi: 10.1093/molbev/msl089 16916942

[B18] Delannoy E. Fujii S. Des Francs-Small C. C. Brundrett M. Small I. (2011). Rampant gene loss in the underground orchid *Rhizanthella gardneri* highlight evolutionary constraints on plastid genomes. Mol. Biol. Evol. 28, 2077–2086. doi: 10.1093/molbev/msr028 21289370 PMC3112369

[B19] Downie S. R. Jansen R. K. (2015). A comparative analysis of whole plastid genomes from the apiales: Expansion and contraction of the inverted repeat, mitochondrial to plastid transfer of DNA, and identification of highly divergent noncoding regions. Syst. Bot. 40, 336–351. doi: 10.1600/036364415X686620

[B20] Doyle J. J. Doyle J. L. Ballenger J. A. Palmer J. D. (1996). The distribution and phylogenetic significance of a 50 kb chloroplast DNA inversion in the flowering plant family leguminosae. Mol. Phylogenet. Evol. 5, 429–438. doi: 10.1006/mpev.1996.0038 8728401

[B21] Feng Y. L. Wicke S. Li J. W. Han Y. Lin C. S. Li D. Z.. (2016). Lineage-specific reductions of plastid genomes in an orchid tribe with partially and fully mycoheterotrophic species. Genome Biol. Evol. 7, 2164–2175. doi: 10.1093/gbe/evw144 PMC4987110 27412609

[B22] Frailey D. C. Chaluvadi S. R. Vaughn J. N. Coatney C. G. Bennetzen J. L. (2018). Gene loss and genome rearrangement in the plastids of five hemiparasites in the family orobanchaceae. BMC Plant Biol. 18, 30. doi: 10.1186/s12870-018-1249-x 29409454 PMC5801802

[B23] Frazer K. A. Pachter L. Poliakov A. Rubin E. M. Dubchak I. (2004). VISTA: computational tools for comparative genomics. Nucleic Acids Res. 32 (suppl_2), W273–W279. doi: 10.1093/nar/gkh458 15215394 PMC441596

[B24] Funk H. T. Berg S. Krupinska K. Maier U. G. Krause K. (2007). Complete DNA sequences of the plastid genomes of two parasitic flowering plant species, *Cuscuta reflexa* and *Cuscuta gronovii* . BMC Plant Biol. 7, 45. doi: 10.1186/1471-2229-7-45 17714582 PMC2089061

[B25] Gao L. Yi X. Yang Y. X. Su Y. J. Wang T. (2009). Complete chloroplast genome sequence of a tree fern *Alsophila spinulosa*: Insights into evolutionary changes in fern chloroplast genomes. BMC Evol Biol. 9, 130. doi: 10.1186/1471-2148-9-130 19519899 PMC2706227

[B26] Goulding S. E. Olmstead R. G. Morden C. W. Wolfe K. H. (1996). Ebb and flow of the chloroplast inverted repeat. Mol. Gen. Genet. 252, 195–206. doi: 10.1007/BF02173220 8804393

[B27] Graham S. W. Lam V. K. Y. Merckx V. S. F. T. (2017). Plastomes on the edge: The evolutionary breakdown of mycoheterotroph plastid genomes. New Phytologist. 214, 48–55. doi: 10.1111/nph.14398 28067952

[B28] Hirao T. Watanabe A. Kurita M. Kondo T. Takata K. (2008). Complete nucleotide sequence of the *Cryptomeria japonica* d. don. chloroplast genome and comparative chloroplast genomics: diversified genomic structure of coniferous species. BMC Plant Biol. 8, 70. doi: 10.1186/1471-2229-8-70 18570682 PMC2443145

[B29] Jansen R. K. Palmer J. D. (1987). Chloroplast DNA from lettuce and *Barnadesia* (Asteraceae): Structure, gene localization, and characterization of a large inversion. Curr. Genet. 11, 553–564. doi: 10.1007/BF00384619

[B30] Jansen R. K. Wojciechowski M. F. Sanniyasi E. Lee S. B. Daniell H. (2008). Complete plastid genome sequence of the chickpea (*Cicer arietinum*) and the phylogenetic distribution of *rps12* and *clpP* intron losses among legumes (*Leguminosae*). Mol. Phylogenet. Evol. 48, 1204–1217. doi: 10.1016/j.ympev.2008.06.013 18638561 PMC2586962

[B31] Jin J. J. Yu W. B. Yang J. B. Song Y. Yi T. S. Li D. Z. (2020). GetOrganelle: A fast and versatile toolkit for accurate *de novo* assembly of organelle genomes. Genome Biol. 21, 241. doi: 10.1101/256479 32912315 PMC7488116

[B32] Kang M. J. Kim S. C. Lee H. R. Lee S. A. Lee J. W. Kim T. D.. (2020). The complete chloroplast genome of Korean *Gastrodia elata* blume. Mitochondrial DNA Part b-resources. 19, 908–917. doi: 10.1080/23802359.2020.1721346 PMC7748522 33366853

[B33] Katoh K. Misawa K. Kuma K. I. Miyata T. (2002). MAFFT: a novel method for rapid multiple sequence alignment based on fast Fourier transform. Nucleic Acids Res. 30, 3059–3066. doi: 10.1093/nar/gkf436 12136088 PMC135756

[B34] Kim Y. K. Jo S. Cheon S. H. Joo M. J. Hong J. R. Kwak M. H.. (2019). Extensive losses of photosynthesis genes in the plastome of a mycoheterotrophic orchid, *Cyrtosia septentrionalis* (Vanilloideae: Orchidaceae). Genome Biol. Evol. 11, 565–571. doi: 10.1093/gbe/evz024 30715335 PMC6390903

[B35] Kim Y. K. Jo S. J. Cheon S. H. Joo M. J. Hong J. R. Kwak M.. (2020). Plastome evolution and phylogeny of orchidaceae, with 24 new sequences. Front. Plant Sci. 11, 1–11. doi: 10.3389/fpls.2020.00022 32153600 PMC7047749

[B36] Kim H. T. Kim J. S. Moore M. J. Neubig K. M. Williams N. H. Whitten W. M.. (2015). Seven new complete plastome sequences reveal rampant independent loss of the *ndh* gene family across orchids and associated instability of the inverted repeat/small single-copy region boundaries. PloS One 10, e0142215. doi: 10.1371/journal.pone.0142215 26558895 PMC4641739

[B37] Kurtz S. Phillippy A. Delcher A. L. Smoot M. Shumway M. Antonescu C.. (2004). Versatile and open software for comparing large genomes. Genome Biol. 5, R12. doi: 10.1186/gb-2004-5-2-r12 14759262 PMC395750

[B38] Lam V. K. Y. Gomez M. S. Graham S. W. (2015). The highly reduced plastome of mycoheterotrophic *Sciaphila* (Triuridaceae) is colinear with its green relatives and is under strong purifying selection. Genome Biol. Evol. 7, 2220–2236. doi: 10.1093/gbe/evv134 26170229 PMC4558852

[B39] Lam V. K. Y. Merckx V. S. F. T. Graham S. W. (2016). A few-gene plastid phylogenetic framework for mycoheterotrophic monocots. Am. J. Bot. 103, 692–708. doi: 10.3732/ajb 27056932

[B40] Leake J. R. (1994). The biology of myco-heterotrophic (‘saprophytic’) plants. New Phytol. 127, 171–216. doi: 10.1111/j.1469-8137.1994.tb04272.x 33874520

[B41] Lee H. L. Jansen R. K. Chumley T. W. Kim K. J. (2007). Gene relocations within chloroplast genomes of *Jasminum* and *Menodora* (Oleaceae) are due to multiple, overlapping inversions. Mol. Biol. Evol. 24, 1161–1180. doi: 10.1093/molbev/msm036 17329229

[B42] Li M. H. Zhang G. Q. Lan S. R. Liu Z. J. (2016). A molecular phylogeny of Chinese orchids. J. Syst. Evol. 54, 349–362. doi: 10.1111/jse.12187

[B43] Lin C. S. Chen J. J. W. Huang Y. T. Chan M. T. Daniell H. Chang W. J.. (2015). The location and translocation of ndh genes of chloroplast origin in the orchidaceae family. Sci. Rep. 5, 1–10. doi: 10.1038/srep09040 PMC4356964 25761566

[B44] Lin C. P. Huang J. P. Wu C. S. Hsu C. Y. Chaw S. M. (2010). Comparative chloroplast genomics reveals the evolution of pinaceae genera and subfamilies. Genome Biol. Evol. 2, 504–517. doi: 10.1093/gbe/evq036 20651328 PMC2997556

[B45] Lohan A. J. Wolfe K. H. (1998). A subset of conserved tRNA genes in plastid DNA of nongreen plants. Genetics 150, 425–433. doi: 10.1046/j.1365-2443.1998.00217.x 9725858 PMC1460314

[B46] Luo J. Hou B. W. Niu Z. T. Liu W. Xue Q. Y. Ding X. Y. (2014). Comparative chloroplast genomes of photosynthetic orchids: insights into evolution of the orchidaceae and development of molecular markers for phylogenetic applications. PloS One 9, e99016. doi: 10.1371/journal.pone.0099016 24911363 PMC4049609

[B47] Merckx V. (2013). Mycoheterotrophy: the biology of plants living on fungi (New York, NY, USA: Springer-Verlag). doi: 10.1007/978-1-4614-5209-6

[B48] Merckx V. Freudenstein J. V. (2010). Evolution of mycoheterotrophy in plants: A phylogenetic perspective. New Phytologist. 185, 605–609. doi: 10.1111/j.1469-8137.2009.03155.x 20356335

[B49] Naumann J. Der J. P. Wafula E. K. Jones S. S. Wagner S. T. Honaas L. A.. (2016). Detecting and characterizing the highly divergent plastid genome of the nonphotosynthetic parasitic plant *Hydnora visseri* (Hydnoraceae). Genome Biol. Evol. 8, 345–363. doi: 10.1093/gbe/evv256 26739167 PMC4779604

[B50] Niu Z. T. Pan J. J. Zhu S. Y. Li L. D. Xue Q. Y. Liu W.. (2017). Comparative analysis of the complete plastomes of *Apostasia wallichii* and *Neuwiedia singapureana* (Apostasioideae) reveals different evolutionary dynamics of IR/SSC boundary among photosynthetic orchids. Front. Plant Sci. 8. doi: 10.3389/fpls.2017.01713 PMC5632729 29046685

[B51] Park J. Suh Y. Kim S. (2020). A complete chloroplast genome sequence of *Gastrodia elata*(Orchidaceae) represents high sequence variation in the species. Mitochondrial Part B-Resources 5, 517–519. doi: 10.1080/23802359.2019.1710588 PMC7748695 33366628

[B52] Petersen G. Cuenca A. Møller M. Seberg O. (2015). Massive gene loss in mistletoe (*Viscum*, viscaceae) mitochondria. Sci. Rep. 5, 17588. doi: 10.1038/srep17588 26625950 PMC4667250

[B53] Poliseno L. Salmena L. Zhang J. W. Carver B. Haveman W. J. Paolo P. (2010). A coding-independent function of gene and pseudogene mRNAs regulates tumour biology. Nature 465, 1033–1038. doi: 10.1038/nature09144 20577206 PMC3206313

[B54] Ravi V. Khurana J. P. Tyagi A. K. Khurana P. (2008). An update on chloroplast genomes. Plant Syst. Evol. 271, 101–122. doi: 10.1007/s00606-007-0608-0

[B55] Roma L. Cozzolino S. Schlüter P. M. Scopece G. Cafasso D. (2018). The complete plastid genomes of *Ophrys iricolor* and *O. sphegodes* (Orchidaceae) and comparative analyses with other orchids. PloS One 13, e0204174. doi: 10.1371/journal.pone.0204174 30226857 PMC6143245

[B56] Sanderson M. J. Copetti D. Búrquez A. Busramante E. Charboneau J. L. M. Eguiarte L. E.. (2015). Exceptional reduction of the plastid genome of saguaro cactus (*Carnegiea gigantea*): Loss of the *ndh* gene suite and inverted repeat. Am. J. Botany 102, 1115–1127. doi: 10.3732/ajb.1500184 26199368

[B57] Saski C. Lee S. B. Daniell H. Wood T. C. Tomkins J. Kim H. G.. (2005). Complete chloroplast genome sequence of *Glycine max* and comparative analyses with other legume genomes. Plant Mol. Biol. 59, 309–322. doi: 10.1007/s11103-005-8882-0 16247559

[B58] Sharp P. M. Li W. H. (1987). The codon adaptation index-a measure of directional synonymous codon usage bias, and its potential applications. Nucleic Acids Res. 15 (3), 1281–1295. doi: 10.1093/nar/15.3.1281 3547335 PMC340524

[B59] Shi L. C. Chen H. M. Jiang M. Wang L. Q. Wu X. Huang L. F.. (2019). CPGAVAS2, an integrated plastome sequence annotator and analyzer. Nucleic Acids Res. 47 (W1), W65–W73. doi: 10.1093/nar/gkz345 31066451 PMC6602467

[B60] Stamatakis A. (2014). RAxML version 8: A tool for phylogenetic analysis and post-analysis of large phylogenies. Bioinformatics 30, 1312–1313. doi: 10.1093/bioinformatics/btu033 24451623 PMC3998144

[B61] Wang H. Harrison S. P. Prentice I. C. Yang Y. Bai F. Togashi H. F.. (2018). The China plant trait database: Toward a comprehensive regional compilation of functional traits for land plants. Ecology 99, 500. doi: 10.1002/ecy.2091 29155446

[B62] Weng M. L. Blazier J. C. Madhumita G. Jansen R. K. (2014). Reconstruction of the ancestral plastid genome in geraniaceae reveals a correlation between genome rearrangements, repeats, and nucleotide substitution rates. Mol. Biol. Evol. 3, 645–659. doi: 10.1093/molbev/mst257 24336877

[B63] Westwood J. H. Yoder J. I. Timko M. P. Depamphilis C. W. (2010). The evolution of parasitism in plants. Trends Plant Sci. 15, 227–235. doi: 10.1016/j.tplants.2010.01.004 20153240

[B64] Wicke S. Müller K. F. Depamphilis C. W. Quandt D. Wickett N. J. Zhang Y.. (2013). Mechanisms of functional and physical genome reduction in photosynthetic and nonphotosynthetic parasitic plants of the broomrape family. Plant Cell. 25, 3711–3725. doi: 10.1105/tpc.113.113373 24143802 PMC3877813

[B65] Wicke S. Müller K. F. Depamphilis C. W. Schneeweiss G. M. (2016). Mechanistic model of evolutionary rate variation en route to a nonphotosynthetic lifestyle in plants. Proc. Natl. Acad. Sci. 113, 9045–9050. doi: 10.1073/pnas.1607576113 27450087 PMC4987836

[B66] Wicke S. Naumann J. (2018). Molecular evolution of plastid genomes in parasitic flowering plants. Adv. Botanical Res. 85, 315–347. doi: 10.1016/bs.abr.2017.11.014

[B67] Wicke S. Schaferhoff B. Depamphilis C. W. Müller K. F. (2014). Disproportional plastome-wide increase of substitution rates and relaxed purifying selection in genes of carnivorous lentibulariaceae. Mol. Biol. Evol. 31, 529–545. doi: 10.1093/molbev/mst261 24344209

[B68] Wicke S. Schneeweiss G. M. Depamphilis C. W. Müller K. F. Quandt D. (2011). The evolution of the plastid chromosome in land plants: Gene content, gene order, gene function. Plant Mol. Biol. 76, 273–297. doi: 10.1007/s11103-011-9762-4 21424877 PMC3104136

[B69] Wolfe K. H. Morden C. W. Palmer J. D. (1992). Function and evolution of a minimal plastid genome from a nonphotosynthetic parasitic plant. Proc. Natl. Acad. Sci. 89, 10648–10652. doi: 10.1073/pnas.89.22.10648 1332054 PMC50398

[B70] Wu C. S. Lai Y. T. Lin C. P. Wang Y. N. Chaw S. M. (2009). Evolution of reduced and compact chloroplast genomes (cpDNAs) in gnetophytes: Selection toward a lower-cost strategy. Mol. Phylogenet. Evol. 52, 115–124. doi: 10.1016/j.ympev.2008.12.026 19166950

[B71] Wu C. S. Wang Y. N. Hsu C. Y. Lin C. P. Chaw S. M. (2011). Loss of different inverted repeat copies from the chloroplast genomes of pinaceae and cupressophytes and influence of heterotachy on the evaluation of gymnosperm phylogeny. Genome Biol. Evol. 3, 1284–1295. doi: 10.1093/gbe/evr095 21933779 PMC3219958

[B72] Yan M. H. Moore M. J. Meng A. P. Yao X. H. (2017). The first complete plastome sequence of the basal asterid family styracaceae (Ericales) reveals a large inversion. Plant Syst. Evol. 303, 61–70. doi: 10.1007/s00606-016-1352-0

[B73] Yang J. B. Tang M. Li H. T. Zhang Z. R. Li D. Z. (2013). Complete chloroplast genome of the genus *Cymbidium*: Lights into the species identification, phylogenetic implications and population genetic analyses. BMC Evol. Biol. 13, 84. doi: 10.1186/1471-2148-13-84 23597078 PMC3644226

[B74] Yang M. Zhang X. W. Liu G. M. Yin Y. X. Chen K. F. Yun Q. Z.. (2010). The complete chloroplast genome sequence of date palm (*Phoenix dactylifera* l.). PloS One 5, e12762. doi: 10.1371/journal.pone.0012762 20856810 PMC2939885

[B75] Yuan Y. Jin X. H. Liu J. Zhao X. Zhou J. H. Wang X.. (2018). The *Gastrodia elata* genome provides insights into plant adaptation to heterotrophy. Nat. Commun. 9, 1615. doi: 10.1038/s41467-018-03423-5 29691383 PMC5915607

[B76] Yue F. Cui L. Y. Depamphilis C. W. Moret B. M. Tang J. J. (2008). Gene rearrangement analysis and ancestral order inference from chloroplast genomes with inverted repeat. BMC Genomics 9, S25. doi: 10.1186/1471-2164-9-S1-S25 PMC2386067 18366615

[B77] Zeng S. Y. Zhou T. Han K. Yang Y. C. Zhao J. H. Liu Z. L. (2017). The complete chloroplast genome sequences of six *Rehmannia* species. Genes 8, 103. doi: 10.3390/genes8030103 28294981 PMC5368707

