# Supplementary material for: Evolutionary differences in gene loss and pseudogenization among mycoheterotrophic orchids in the tribe Vanilleae (subfamily Vanilloideae)
Source: Front Plant Sci. 2023 Mar 22;14:1160446. doi: 10.3389/fpls.2023.1160446 (PMC10073425; doi:10.3389/fpls.2023.1160446)
Supplement: Supplementary file 1 [file DataSheet_1.pdf]

Table S1. Copy number variations of TR units in *G. lindleyana* chloroplast genome

No.	TR Unit Sequence	Unit Length (bp)	Copy Number	Position	Percent Matches	Percent Indels	A %	T %	C %	G %	gene position
**1**	CTATATA	7	10	LSC	0.76	0.21	0.48,	0.4	0.10,	0.03,	
**2**	TATAGTAT	8	10.2	LSC	0.73	0.17	0.36,	0.51	0.01,	0.12,	
**3**	ATATATATA	9	4	SSC	0.93	0	0.53,	0.47	0.00,	0.00,	
**4**	TTATATATA	9	5.1	LSC	0.79	0.21	0.43,	0.55	0.00,	0.02,	
**5**	TATTATATT	9	15.3	LSC	0.67	0.31	0.31,	0.68	0.00,	0.02,	
**6**	TAAATAAAAA	10	5.3	LSC	0.74	0.22	0.73,	0.25	0.00,	0.02,	
**7**	AATAAATATA	10	6.2	LSC	0.74	0.23	0.69,	0.28	0.02,	0.02,	
**8**	ATAAAAAGGAT	11	2.7	LSC	0.95	0	0.63,	0.2	0.00,	0.17,	*psb*C 35645-36656
**9**	AAAAACTATAG	11	2.9	LSC	0.95	0.05	0.68,	0.19	0.10,	0.03,	
**10**	TTTTATTCTTA	11	3.5	LSC	0.86	0.1	0.18,	0.77	0.05,	0.00,	
**11**	ATATATATCTA	11	3.5	LSC	0.9	0.07	0.50,	0.42	0.07,	0.00,	
**12**	TAAAGTATAAA	11	4.8	LSC	0.76	0.24	0.61,	0.31	0.00,	0.08,	
**13**	ATATATATATT	11	8.9	SSC	0.74	0.22	0.45,	0.51	0.00,	0.04,	
**14**	TTTATACTTCAA	12	2.2	LSC	1	0	0.30,	0.56	0.15,	0.00,	
**15**	CAGTTACATTTA	12	2.4	LSC	1	0	0.31,	0.41	0.17,	0.10,	*acc*D 42133-43743
**16**	ATAGTAGTCAAT	12	2.7	LSC	0.95	0	0.38,	0.34	0.09,	0.19,	
**17**	TTTTATTCTTAT	12	3.2	LSC	0.81	0.15	0.16,	0.78	0.05,	0.00,	
**18**	TTTTATTTTTTA	12	3.7	LSC	0.86	0.09	0.20,	0.78	0.00,	0.02,	
**19**	ATATACTATATA	12	3.7	LSC	1	0	0.50,	0.41	0.09,	0.00,	
**20**	ATATATAACTAA	12	4.4	LSC	0.88	0.05	0.51,	0.4	0.08,	0.02,	
**21**	GTCTTAGTATTA	12	5.2	SSC	0.73	0.14	0.30,	0.48	0.06,	0.16,	
**22**	AATTATAAGATG	12	5.4	LSC	0.7	0.23	0.56,	0.34	0.00,	0.10,	
**23**	TAAATATAGAAA	12	5.8	LSC	0.7	0.17	0.60,	0.3	0.04,	0.06,	
**24**	TATATATAAGTTA	13	2	LSC	1	0	0.46,	0.46	0.00,	0.08,	
**25**	TTGTTATATAATA	13	2.1	LSC	1	0	0.37,	0.56	0.00,	0.07,	
**26**	AATAAAAATAAAT	13	2.5	LSC	0.89	0.11	0.77,	0.23	0.00,	0.00,	
**27**	TTTTATTTTCCTT	13	2.7	LSC	0.88	0.12	0.11,	0.78	0.11,	0.00,	
**28**	ATAGTATTAATTA	13	3	LSC	0.77	0.23	0.44,	0.47	0.00,	0.08,	
**29**	ATATATATAATAT	13	3.2	LSC	0.89	0.07	0.51,	0.46	0.03,	0.00,	

**30**	ATATTATATATAC	13	3.8	LSC	0.84	0.05	0.44,	0.5	0.04,	0.02,		
**31**	TAAGTTAAGTATAT	14	2	LSC	1	0	0.43,	0.43	0.00,	0.14,		
**32**	AGTAATCAAGTAAA	14	2.1	LSC	1	0	0.59,	0.21	0.07,	0.14,		
**33**	TATAGTATAATAAA	14	2.1	LSC	0.94	0.06	0.59,	0.34	0.00,	0.07,		
**34**	TTTCCATATCTATA	14	2.1	SSC	1	0	0.28,	0.52	0.21,	0.00,		
**35**	TTATACTACTACTA	14	6.1	SSC	0.85	0.12	0.36,	0.43	0.21,	0.00,		
**36**	TTAGAATAGATTTAT	15	2.1	LSC	0.94	0	0.39,	0.52	0.00,	0.10,		
**37**	TATTTAAAATTAAAT	15	2.1	LSC	1	0	0.52,	0.48	0.00,	0.00,		
**38**	CTAAGGAATCATTAA	15	2.3	SSC	0.9	0.05	0.44,	0.29	0.12,	0.15,	*ycf*1	76186-81600
**39**	TGTATCTTCGTTATA	15	2.3	SSC	0.9	0	0.17,	0.57	0.14,	0.11,	*ycf*1	76186-81600
**40**	TTTATTTTAATTACT	15	2.9	LSC	0.87	0.13	0.24,	0.69	0.07,	0.00,		
**41**	TCTATCTTTACTTAC	15	3	LSC	0.81	0.06	0.16,	0.56	0.29,	0.00,		
**42**	ATATGTATATATTAT	15	7.1	LSC	0.76	0.15	0.39,	0.52	0.02,	0.07,		
**43**	TTATATATTTTTATAC	16	1.9	LSC	0.93	0	0.29,	0.65	0.03,	0.03,		
**44**	AATAGTATTAATTATG	16	1.9	LSC	0.93	0.07	0.47,	0.43	0.00,	0.10,		
**45**	ATATAAAATAAAGGAA	16	1.9	LSC	0.93	0	0.65,	0.19	0.00,	0.16,		
**46**	TAGTTAATATCCTATG	16	2.1	LSC	1	0	0.32,	0.44	0.12,	0.12,		
**47**	TTCAATTCCATAATTA	16	2.4	LSC	0.91	0	0.33,	0.51	0.15,	0.00,		
**48**	TGTATATTTACATTTA	16	2.4	LSC	0.83	0.13	0.30,	0.62	0.03,	0.05,		
**49**	AATTTATTAATATTTA	16	3	LSC	0.74	0.26	0.44,	0.54	0.02,	0.00,		
**50**	ATAAGAATATAGAAGA	16	3.2	LSC	0.89	0.11	0.65,	0.19	0.00,	0.17,		
**51**	ATATAAAAATAAATAT	16	3.8	LSC	0.78	0.14	0.66,	0.26	0.00,	0.08,		
**52**	TATAATTAAATATAAT	16	3.9	LSC	0.75	0.2	0.59,	0.41	0.00,	0.00,		
**53**	TATAGTATTATATAGT	16	4.8	LSC	0.83	0.11	0.36,	0.51	0.01,	0.12,		
**54**	TATTATTTTATATTAT	16	7.9	LSC	0.77	0.14	0.31,	0.68	0.00,	0.02,		
**55**	ATATATATGGATATGTC	17	2.1	LSC	0.89	0	0.34,	0.43	0.09,	0.14,		
**56**	TAGATTAGAATCTAATA	17	2.9	LSC	0.76	0.24	0.46,	0.39	0.04,	0.11,		
**57**	ATAATATTAAATTTATA	17	3.8	LSC	0.79	0.08	0.47,	0.49	0.01,	0.03,		
**58**	ATTTATATAGTAGATATT	18	2.1	LSC	0.95	0	0.39,	0.53	0.00,	0.08,		
**59**	TTTTTATTTATATTATTA	18	2.1	LSC	1	0	0.27,	0.73	0.00,	0.00,		
**60**	TATTTCGATTTTAATTTA	18	2.1	LSC	0.89	0.05	0.25,	0.67	0.03,	0.06,		
**61**	AGTAAGGAAAACACATCA	18	2.1	IRB	0.89	0	0.57,	0.14	0.14,	0.16,		
**62**	TTGATGTCTTTTCATTAC	18	2.1	IRA	0.89	0	0.14,	0.57	0.16,	0.14,		

**63**	TATTTATATTATTATTTA	18	2.2	LSC	0.91	0.04	0.32,	0.68	0.00,	0.00,		
**64**	AATATGAATATAATAATG	18	2.2	LSC	0.9	0	0.54,	0.36	0.03,	0.08,		
**65**	TTTTAGTTTATTATTTTA	18	2.2	LSC	0.9	0.05	0.18,	0.79	0.00,	0.03,		
**66**	TATATTAGATACTAGAAT	18	2.3	LSC	0.91	0	0.41,	0.44	0.07,	0.07,		
**67**	AAATAATAATAAATAGAA	18	2.5	LSC	0.83	0.1	0.74,	0.23	0.00,	0.02,		
**68**	AAGAAGAATAAGAATATG	18	2.8	LSC	0.76	0.21	0.65,	0.19	0.00,	0.17,		
**69**	AATGATTTTGATAGCTCT	18	11.1	LSC	0.9	0.04	0.30,	0.42	0.12,	0.16,	*acc*D	42133-43743
**70**	AATAAGTTCTAAATTACTT	19	2.1	LSC	0.95	0	0.45,	0.42	0.10,	0.03,		
**71**	TATTTATAGATTCTATATC	19	2.4	LSC	0.8	0.2	0.33,	0.54	0.09,	0.04,		
**72**	TTTACTTACTCTATTTCTC	19	2.7	LSC	0.74	0.18	0.17,	0.59	0.24,	0.00,		
**73**	AGTCTTAGTATTAGTTATA	19	3	SSC	0.97	0	0.33,	0.47	0.05,	0.14,		
**74**	TATATATATATAATATACA	19	3.3	LSC	0.93	0.07	0.51,	0.44	0.05,	0.00,		
**75**	TTCATATAGCATAGCATATA	20	2	LSC	1	0	0.40,	0.35	0.15,	0.10,		
**76**	TATAGAATATTCTATATAGA	20	2.1	LSC	0.96	0	0.44,	0.44	0.05,	0.07,		
**77**	TGAAATAAAAAATACTTAGA	20	2.9	LSC	0.74	0.21	0.59,	0.27	0.05,	0.08,		
**78**	ATTTTAATTTTATTTCCCTCT	21	1.9	SSC	0.89	0.05	0.18,	0.64	0.18,	0.00,		
**79**	CTATACAAAAACTATAAAAAA	21	2.3	LSC	0.79	0.1	0.61,	0.2	0.14,	0.04,		
**80**	TATATATTTTATTTCAATTAT	21	2.4	LSC	0.94	0.03	0.35,	0.62	0.04,	0.00,		
**81**	TTTCTATATTTATATTTTAGA	21	2.9	SSC	0.88	0.1	0.29,	0.62	0.05,	0.03,		
**82**	ATAAATTATAAGATGAATTAA	21	3.2	LSC	1	0	0.59,	0.32	0.00,	0.09,		
**83**	TTTATATTTCTTTCTTTCATT	21	3.4	LSC	0.67	0.25	0.18,	0.69	0.11,	0.03,		
**84**	TAATAGGATACATTTTATATAT	22	2	LSC	0.87	0	0.36,	0.51	0.04,	0.09,		
**85**	ATATAAATGAATACATAAATATA	23	2.3	LSC	0.87	0.1	0.62,	0.3	0.04,	0.04,		
**86**	ATATAAGAATAAGAAGAAGAAGA	23	2.4	LSC	0.83	0.11	0.63,	0.21	0.00,	0.16,		
**87**	ATATAAATATAAATTAAATAGAT	23	3	LSC	0.78	0.12	0.65,	0.31	0.01,	0.03,		
**88**	TTATTATAGTATTATAGTCTTAA	23	3.2	LSC	0.85	0.13	0.38,	0.52	0.01,	0.08,		
**89**	TAATATTTATATTTAAAATTAAAT	24	2.1	LSC	0.85	0.07	0.48,	0.52	0.00,	0.00,		
**90**	ATATATATATCTAATATATAACTA	24	2.4	LSC	0.86	0.06	0.52,	0.4	0.07,	0.02,		
**91**	TTATATTTATATTTACATAATTTA	24	2.6	LSC	0.86	0.1	0.38,	0.6	0.02,	0.00,		
**92**	ATAGATAGGACTAAGAAATAGTAA	24	2.8	LSC	0.9	0	0.52,	0.21	0.05,	0.23,		
**93**	TCATAAAGATCGGCACCCTCAATC	24	3.2	IRA	0.75	0.11	0.33,	0.25	0.29,	0.13,	*ycf*2	91020-97907
**94**	CGATCTTTATGATAGTGACGATAG	24	3.8	IRB	0.76	0.06	0.27,	0.32	0.13,	0.28,		
**95**	TCACTATCATAAAGATCGCTATCG	24	3.8	IRA	0.76	0.06	0.32,	0.27	0.28,	0.13,	*ycf*2	91020-97907

**96**	TATTTATATCTTTATTTATATATAGA	26	2.8	SSC	0.82	0.08	0.33,	0.63	0.03,	0.01,		
**97**	TTTATATTTATATATTTATTTCTTTA	26	2.8	SSC	0.87	0.02	0.32,	0.64	0.03,	0.01,		
**98**	AATATCATATAAATATAAATTAAATAG	27	3	LSC	0.75	0.24	0.64,	0.31	0.03,	0.03,		
**99**	AATAAATAAAATATAAATAAATAAATAA	28	2.2	LSC	0.79	0.13	0.69,	0.28	0.02,	0.02,		
**100**	TATATCTATATCTAGATATAGATATATTTA	30	1.9	LSC	0.93	0.07	0.39,	0.47	0.07,	0.07,		
**101**	TAAGAAGAATAAGAATATAAAGAATAAGAA	30	2.4	LSC	0.84	0.11	0.62,	0.22	0.01,	0.14,		
**102**	ATATACTATTATACATAATATACTATACAT	30	2.5	LSC	0.78	0.13	0.47,	0.44	0.10,	0.00,		
**103**	ATATATATAAATAGAAGTAGAAGTATATATGT	32	1.9	LSC	0.9	0.03	0.49,	0.37	0.00,	0.14,		
**104**	TTATATTTATTTATATATATTAAATCTTAATC	32	2.7	LSC	0.78	0.19	0.36,	0.59	0.05,	0.00,		
**105**	TTATATTTATTTATATATATTATATCTTTATC	32	3.5	LSC	0.74	0.21	0.34,	0.6	0.05,	0.01,		
**106**	CTATATATTTGTATTTCAGATTTCTATATATTT	33	2	LSC	0.85	0.12	0.30,	0.61	0.06,	0.03,		
**107**	GTATATTATGCATATATATAGAATAGAAGTAGAA	34	2.2	LSC	0.8	0.12	0.47,	0.38	0.01,	0.14,		
**108**	TAATATAAATAATATAATTATAATTAAATATAAA	34	2.7	LSC	0.78	0.14	0.61,	0.38	0.00,	0.01,		
**109**	TATATCTTTATTTAATTTAATTATATACTATATAAAT	37	2.7	LSC	0.77	0.13	0.36,	0.58	0.06,	0.00,		
**110**	TATTTTTTTATTATTATGTTTATATTATTATTAATTAT	38	3.3	LSC	0.86	0.1	0.31,	0.68	0.00,	0.02,		
**111**	AAATATAGATAAAAGATAAATGATATATAAAAATAAAGG	39	1.9	LSC	0.82	0.13	0.64,	0.23	0.01,	0.12,		
**112**	ATATAGATATTTATATCCTTATTTATATATATATATTTAT	40	3.1	SSC	0.79	0.12	0.34,	0.61	0.04,	0.02,		
**113**	TATATATATATTTTAGATATACATATATATATCTTTATCTATA TT	45	2.3	SSC	0.83	0.03	0.33,	0.61	0.04,	0.02,		
**114**	ATGAGATTGAGGGTGCCGATCTTTATGATAGTGACGATAGC GATCTTT	48	2	IRB	0.96	0.04	0.25,	0.33	0.14,	0.28,		
**115**	AAGATCGCTATCGTCACTATCATAAAGATCGGCACCCTCAAT CTCATA	48	2	IRA	0.96	0.04	0.33,	0.25	0.28,	0.14,	*ycf*2	91020-97907
**116**	TAACTTTATCTTACTTAATTATCTATAAGTACTCCCATTTTCC TATCTTTCCTCTTTATATGA	63	2	SSC	0.98	0	0.25,	0.5	0.21,	0.03,		
