# Supplementary figures and images for: Evolutionary differences in gene loss and pseudogenization among mycoheterotrophic orchids in the tribe Vanilleae (subfamily Vanilloideae)
Source: Front Plant Sci. 2023 Mar 22;14:1160446. doi: 10.3389/fpls.2023.1160446 (PMC10073425; doi:10.3389/fpls.2023.1160446)

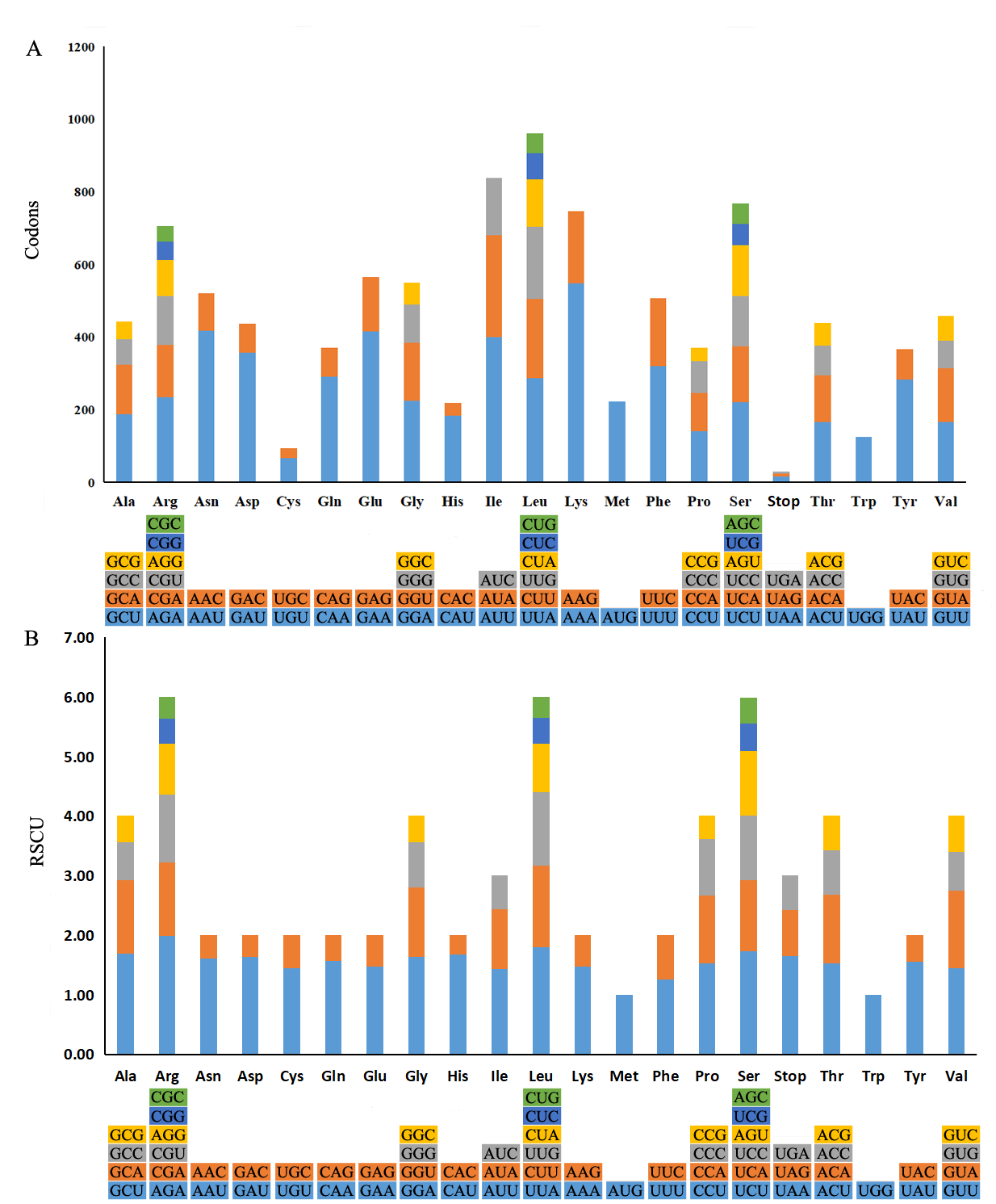

Supplement: Supplementary Figure 1 — Codon frequencies and RSCU values of G. lindleyana: (A) Amino acid frequencies in protein-coding genes; (B) RSCU values of 20 amino acids and stop codons in 32 protein-coding genes. [file Image_1.tif]
